# GENOMES UNCOUPLED PROTEIN1 binds to plastid RNAs and promotes their maturation

**DOI:** 10.1016/j.xplc.2024.101069

**Published:** 2024-08-22

**Authors:** Qian Tang, Duorong Xu, Benjamin Lenzen, Andreas Brachmann, Madhura M. Yapa, Paymon Doroodian, Christian Schmitz-Linneweber, Tatsuru Masuda, Zhihua Hua, Dario Leister, Tatjana Kleine

**Affiliations:** 1 Plant Molecular Biology (Botany), Faculty of Biology, Ludwig-Maximilians-University München, 82152 Martinsried, Germany; 2 Molecular Genetics, Humboldt-University Berlin, Philippstr. 13, 10115 Berlin, Germany; 3 Biocenter of the LMU Munich, Genetics Section, Grosshaderner Str. 2-4, 82152 Planegg-Martinsried, Germany; 4 Department of Environmental and Plant Biology, Ohio University, Athens, OH 45701, USA; 5 Graduate School of Arts and Sciences, The University of Tokyo, Komaba, Meguro-ku 153-8902, Tokyo, Japan

**Keywords:** GUN1, MORF2, plastid (post)transcriptome, retrograde signaling, RIP-seq, RNA binding protein

## Abstract

Plastid biogenesis and the coordination of plastid and nuclear genome expression through anterograde and retrograde signaling are essential for plant development. GENOMES UNCOUPLED1 (GUN1) plays a central role in retrograde signaling during early plant development. The putative function of GUN1 has been extensively studied, but its molecular function remains controversial. Here, we evaluate published transcriptome data and generate our own data from *gun1* mutants grown under signaling-relevant conditions to show that editing and splicing are not relevant for GUN1-dependent retrograde signaling. Our study of the plastid (post)transcriptome of *gun1* seedlings with white and pale cotyledons demonstrates that GUN1 deficiency significantly alters the entire plastid transcriptome. By combining this result with a pentatricopeptide repeat code-based prediction and experimental validation by RNA immunoprecipitation experiments, we identified several putative targets of GUN1, including tRNAs and RNAs derived from *ycf1.2*, *rpoC1*, and *rpoC2* and the *ndhH*–*ndhA*–*ndhI*–*ndhG*–*ndhE*–*psaC*–*ndhD* gene cluster. The absence of plastid rRNAs and the significant reduction of almost all plastid transcripts in white *gun1* mutants account for the cotyledon phenotype. Our study provides evidence for RNA binding and maturation as the long-sought molecular function of GUN1 and resolves long-standing controversies. We anticipate that our findings will serve as a basis for subsequent studies on mechanisms of plastid gene expression and will help to elucidate the function of GUN1 in retrograde signaling.

## Teaser

GENOMES UNCOUPLED1 (GUN1) plays a central role in retrograde signaling during early plant development. Although it has been extensively studied, its molecular function has remained controversial. This study provides evidence that GUN1 is an RNA-binding protein that promotes plastid RNA maturation, providing a foundation for future studies of plastid gene expression and the function of GUN1 in retrograde signaling.

## Introduction

Chloroplasts are the characteristic organelles of algae and plants, and it is generally accepted that they are derived from ancient cyanobacteria through endosymbiosis (Archibald, 2015). During evolution, most genes of the endosymbiont were transferred to the nuclear genome, resulting in only about 100 genes being present in current plastid genomes (Kleine et al., 2009) and at least 3000 plastid proteins being encoded in the nucleus (Christian et al., 2020). As a result, most plastid multiprotein complexes, such as the plastid gene expression (PGE) machinery and the photosynthetic apparatus, are formed by a mixture of plastid- and nuclear-encoded proteins, requiring coordination of the expression of both genomes. Because most plastid proteins are encoded in the nucleus, this organelle exerts anterograde control over the plastids. For example, the process of PGE necessitates the involvement of diverse nuclear-encoded proteins that promote the transcription, splicing, trimming, and editing of RNA in organelles while simultaneously regulating their translation (Borner et al., 2015; Kleine and Leister, 2015; Small et al., 2023; Zhang et al., 2023). On the other hand, nuclear gene expression, such as expression of the so-called photosynthesis-associated nuclear genes (PhANGs), is controlled by plastid-to-nucleus retrograde signaling (Kleine and Leister, 2016; Liebers et al., 2022), which is thought to be mediated by multiple factors and sources. For instance, in seedlings treated with norflurazon (NF) or lincomycin (LIN), mRNA levels of *PhANGs* are repressed (Oelmuller et al., 1986). NF is an inhibitor of carotenoid biosynthesis (Oelmuller et al., 1986), whereas LIN targets peptidyl transferase domain V of the 23S ribosomal RNA (rRNA) of the 50S ribosomal subunit, which is the site of peptide bond formation, thereby preventing peptide bond formation (Hong et al., 2014). A mutant screen with *Arabidopsis thaliana* (*Arabidopsis* hereafter) identified a group of *genomes uncoupled* (*gun*) mutants three decades ago (Susek et al., 1993). In these mutants, expression of the *PhANG*s, in particular the marker gene *LHCB1.2*, which encodes a light-harvesting chlorophyll a/b-binding protein of photosystem (PS) II, is de-repressed in seedlings treated with an inhibitor (Susek et al., 1993). The original gun screens (Susek et al., 1993; Woodson et al., 2011) led to discovery of six *gun* mutants, five of which, *gun2* to *gun6*, are impaired in the tetrapyrrole biosynthesis pathway. The *gun1* mutant exhibits a distinct *gun* phenotype when treated with LIN, distinguishing it from the other mutants (summarized in Richter et al., 2023). *GUN1* encodes a chloroplast pentatricopeptide repeat (PPR) protein (Koussevitzky et al., 2007). PPR proteins belong to a large family, with an estimated 106 of these proteins targeted to chloroplasts (Small et al., 2023). They participate in various PGE steps, including RNA cleavage, splicing, editing, stabilization, and translation (Small et al., 2023; Zhang et al., 2023). Thus far, no other *ppr* mutant has been identified as a *gun* mutant, indicating that GUN1 is a special component of an anterograde–retrograde axis.

GUN1 is an ancient protein that evolved within the streptophyte clade of the algal ancestors of land plants before the first plants colonized land more than 470 million years ago. It has been suggested that the primary role of GUN1 is to act in PGE and that its involvement in retrograde signaling probably evolved more recently (Honkanen and Small, 2022). In fact, GUN1 contains two domains known to interact with nucleic acids, the PPR domain and a MutS-related (SMR) domain (Koussevitzky et al., 2007). Among a large number of PPR proteins, *Arabidopsis* contains only eight PPR-SMR proteins, five of which are predicted to be localized in chloroplasts (Zhang and Lu, 2019), including PLASTID TRANSCRIPTIONALLY ACTIVE 2, SUPPRESSOR OF VARIEGATION 7 (SVR7), EMBRYO DEFECTIVE 2217, SUPPRESSOR OF THYLAKOID FORMATION 1 (SOT1), and GUN1. Mutants of the first four show severe molecular and/or visible phenotypes, but only SOT1 has been shown to have an RNA-binding function (Zhou et al., 2017; Zhang and Lu, 2019). Mainly by studying *gun1* seedlings grown on inhibitors or in combination with other mutants, GUN1 has been implicated in a variety of processes in chloroplasts, such as regulation of tetrapyrrole biosynthesis (Shimizu et al., 2019), protein homeostasis (Tadini et al., 2016), ribosome maturation (Paieri et al., 2018), accumulation of certain chloroplast transcripts, and chloroplast import (Tadini et al., 2020), to name a few. Recently, GUN1 has been proposed to cooperate with MULTIPLE ORGANELLAR RNA EDITING FACTOR 2 (MORF2)/DIFFERENTIATION AND GREENING-LIKE 1 to regulate RNA editing under NF conditions (Zhao et al., 2019). In the suggested mechanism, GUN1 would not bind directly to the target RNAs. Rather, it would facilitate differential editing through its interaction with MORF2. Although GUN1 has been suggested to interact with DNA *in vitro* (Koussevitzky et al., 2007), no function in nucleic acid binding has yet been demonstrated *in vivo*, although the hypothesis that GUN1 exerts its function by binding RNA has recently been illuminated (Loudya et al., 2024). Furthermore, apart from occasional observations of pale cotyledons in a proportion of seedlings (e.g., in Ruckle et al., 2007), no clear severe phenotype has been observed.

In this study, we revisit the editing functions of GUN1 and MORF2 during retrograde signaling, define a distinct *gun1* phenotype with white cotyledons but green true leaves, examine the *gun1* (post)transcriptome in detail, and perform RNA immunoprecipitation (RIP) and electrophoretic shift experiments that strongly suggest an RNA-binding function of GUN1.

## Results

### GUN1 does not play a significant role in plastid RNA editing or splicing during retrograde signaling

**Figure 1:**
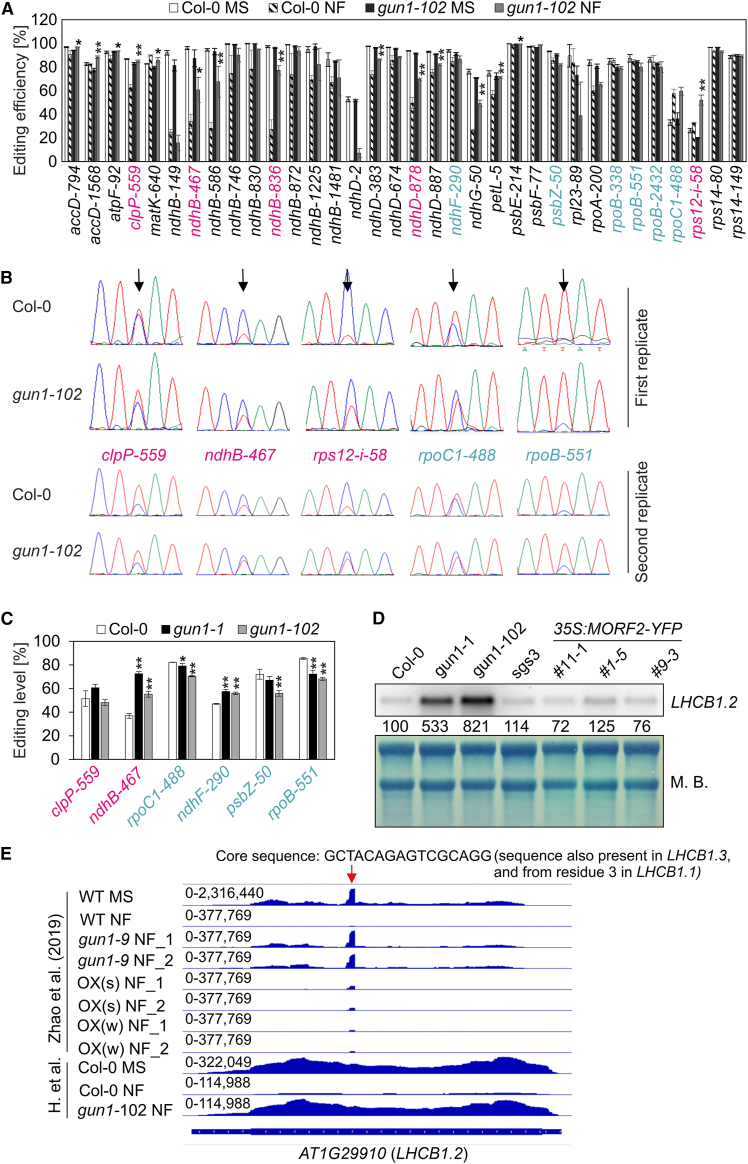
GUN1 does not play a significant role in plastid RNA editing or splicing during retrograde signaling. **(A)** RNA editing efficiencies of 4-day-old Col-0 and *gun1-102* seedlings grown on MS and norflurazon (NF) were determined using previously published RNA-seq data (Habermann et al., 2020). These sequencing data were generated to allow for the detection of organellar transcripts. Mean values ± standard deviations were obtained from three independent experiments. Statistically significant differences between Col-0 NF and *gun1-102* NF are indicated (post hoc Tukey’s HSD [honestly significant difference] test; ∗*P* < 0.05 and ∗∗*P* < 0.01). A graph showing the statistical differences between Col-0 MS and *gun1-102* MS can be found in Supplemental Figure 1. The efficiency of editing sites labeled in magenta and turquoise was found to be elevated and reduced, respectively, by Zhao et al. (2019). We also identified an unexpected increase in editing of *rpoC1* in both WT and *gun1-102* under NF treatment. Our results may vary due to the use of different analysis methods—Sanger sequencing versus lncRNA-seq data analysis—as well as discrepancies in growth media and conditions. Notably, Zhao et al. (2019) cultivated 5-day-old seedlings on MS plates without sucrose, whereas Habermann et al. (2020) used MS plates with 1.5% sucrose. Thus, to account for these variations, we repeated the experiment for selected editing sites in two distinct laboratories as shown in **(B)** and **(C)**. **(B)** Col-0 and *gun1-102* seedlings were grown in laboratory 1 for 5 days under continuous light conditions as reported by Zhao et al. (2019). The editing efficiency of the selected sites was visualized by Sanger sequencing for two biological replicates. **(C)** Col-0, *gun1-1*, and *gun1-102* seedlings were grown in laboratory 2 for 5 days under continuous light conditions as reported by Zhao et al. (2019). The editing efficiency of the selected sites was determined by amplicon sequencing. Mean values with their standard deviations are shown. Statistically significant differences between Col-0 and *gun1* seedlings are indicated (post hoc Tukey’s HSD test; ∗*P* < 0.05 and ∗∗*P* < 0.01). **(D)** Overexpression of MORF2 does not result in a significant *gun* phenotype. Steady-state levels of *LHCB1.2* transcripts in 5-day-old seedlings grown under NF conditions are shown. Col-0 serves as the WT control for *gun1* and *sgs3-1* as a control for oeMORF2 (*35S:MORF2-YFP*) lines. For each genotype, the total RNA was fractionated on a formaldehyde-containing denaturing gel, transferred to a nylon membrane, and probed with [α-^32^P]dCTP-labeled complementary DNA (cDNA) fragments specific for the transcripts encoding *LHCB1.2*. rRNA was visualized by staining the membrane with methylene blue (M.B.) and served as a loading control. Quantification of signals relative to the WT (=100) is provided below each lane. **(E)** Snapshots of reanalyzed RNA-seq data published by Zhao et al. (2019) and Habermann et al. (2020). The read depths were visualized with the Integrated Genome Browser. Whereas reads from Habermann et al. (2020) are evenly distributed across *LHCB1.2*, reads generated by Zhao et al. (2019) exhibit a prominent peak of 16 nucleotides (red arrow). The sequence of the peak (5′-GCTACAGAGTCGCAGG-3′) is also present in *LHCB1.3* and from the third nucleotide in *LHCB1.1*. The sequence of this peak coincides with the sequence of the “LHB1.2” forward primer (actually detecting *LHCB1.3* in combination with the given reverse primer) used by Zhao et al. (2019) for RT–qPCR.

On the basis of Sanger sequencing data analysis, GUN1 has been proposed to regulate plastid RNA editing during retrograde signaling (Zhao et al., 2019). Previously, RNA sequencing after rRNA depletion (long non-coding RNA sequencing [lncRNA-seq]) data covering both nuclear and organellar transcripts were generated for wild-type (WT) and *gun1-102* seedlings grown on Murashige and Skoog (MS) and NF (Habermann et al., 2020). The benefit of the lncRNA-seq technique is that its workflow involves library preparation after depletion of rRNAs rather than enrichment of mRNAs, the latter approach having been used in Zhao et al. (2019) and many other studies analyzing *gun1* mutants. Analysis of the sequences generated by Habermann et al. (2020) for splicing and editing changes revealed no significant alterations between WT and *gun1-102* when grown on MS (Supplemental Figures 1A and 1B). NF had a significant (secondary) effect on plastid splicing, which was similarly reduced in WT and *gun1-102* (Supplemental Figure 1C). Also, no major differences in editing (C-to-U base substitutions) efficiencies were observed between *gun1-102* and WT grown on MS (Supplemental Figure 1D), consistent with previous findings (Zhao et al., 2019). Editing was reduced at multiple sites in NF-treated WT (Figure 1A), confirming that editing is altered under stress exposure (Kakizaki et al., 2009; Zhao et al., 2019). According to Zhao et al. (2019), GUN1-mediated editing is particularly important under inhibitor treatment. They found that RNA editing levels in *gun1-8* and *gun1-9* increased for *clpP-559*, *ndhB-467*/*-836*, *ndhD-878*, and *rps12-i-58* but decreased for *rpoC1-488*, *ndhF-290*, *psbZ-50*, and *rpoB-338/-551/-2432* compared with the WT when grown on NF. We confirmed increased editing levels in *gun1-102* for the same sites (Figure 1A) but observed only a moderate reduction in RNA editing at two sites, *psbZ-50* (87% in WT, 82% in *gun1-102*) and *rpoB-338* (87% in WT, 79% in *gun1-102*). To account for the different growth and analysis conditions, we repeated the experiment in two different laboratories using the growth conditions employed by Zhao et al. (2019). Laboratory 1 used *gun1-102* in Sanger sequencing experiments (Figure 1B), and laboratory 2 included both *gun1-1* and *gun1-102* in amplicon sequencing experiments (Figure 1C). These experiments revealed no reproducible differences in editing efficiency between WT and *gun1* under NF conditions except for a slight reduction in *rpoC1-488* and *rpoB-551* editing.

To summarize, the presence of only mild editing and splicing differences between WT and *gun1* upon NF treatment argue against a major impact of these processes in GUN1 signaling.

### Overexpression of MORF2 does not result in a significant *gun* phenotype

Previously, two MORF2 overexpression lines, *MORF2OX(s)* and *MORF2OX(w)*, were constructed (Zhao et al., 2019). *MORF2OX(s)* exhibited a *gun* phenotype, as its mRNA levels of nuclear-encoded photosynthesis genes, including *LIGHT HARVESTING CHLOROPHYLL A/B BINDING PROTEIN1.2* (*LHCB1.2*), were higher than those of the WT when the seedlings were treated with NF (Zhao et al., 2019). We found that overexpression of *MORF2* in the Col-0 background induced co-suppression of *MORF2* and led to variegation phenotypes in both early seedlings and adult plants (Yapa et al., 2023), similar to those observed for *MORF2OX(s)* (Zhao et al., 2019). To prevent potential post-transcriptional co-suppression-mediated gene silencing, we introduced a *35S:MORF2-YFP* construct into *suppressor of gene silencing 3-1* (*sgs3-1*) plants (Peragine et al., 2004) (Supplemental Figure 2). At the cotyledon stage, lines *35S-MORF2-YFP #1-5* and *#11-1* exhibited phenotypes similar to those of Col-0 and *sgs3-1*. However, line *#9-3*, which had the highest induction of *MORF2* levels (Supplemental Figure 2A), displayed a reduction in the maximum quantum yield of PSII (measured as the parameter Fv/Fm) (Supplemental Figure 2B). The determination of editing levels for *ndhF-290*, *psbZ-50*, *rpoB-338*, and *rpoB-551*, sites that have been described as less edited in both *MORF2OX(s)* and *gun1–9* seedlings under NF treatment (Zhao et al., 2019), indicated that, interestingly, the editing levels of *ndhF-290* and *psbZ-50* were also compromised in our strongest *MORF2 overexpressor (#9-3) (*Supplemental Figure 3A) compared with its parent plant, *sgs3-1* (Supplemental Figure 3B).

To examine the *gun* phenotype of *35S:MORF2-YFP* lines, RT–qPCR was performed on retrograde marker genes. As expected, mRNA levels of the marker genes *LHCB1.2*, *CARBONIC ANHYDRASE 1*, and *PLASTOCYANIN* were higher in the *gun1* alleles. Although *LHCB1.2*, *CARBONIC ANHYDRASE 1*, and *PLASTOCYANIN* mRNA levels were slightly elevated in line *#9-3*, they remained significantly lower than in *gun1* mutants and similar to those in *sgs3-1* (Supplemental Figure 3C). Also, northern blot analysis showed high levels of *LHCB1.2* in *gun1* alleles but WT-like levels in the *35S:MORF2-YFP* lines (Figure 1D). We reanalyzed RNA-seq data generated for WT, *gun1-9*, *oeMORF2(s)*, and *oeMORF2(w)* (Zhao et al., 2019) and sequencing data from Habermann et al. (2020) and plotted the reads across the *LHCB1.2* gene. Whereas the data from Habermann et al. (2020) showed an even distribution of reads across *LHCB1.2*, the reads generated by Zhao et al. (2019) exhibited a prominent peak of 16 nucleotides (Figure 1E). It is predominantly this peak that is found in MORF2 overexpressors after NF treatment (Zhao et al., 2019), whereas there are almost no reads for the remainder of the *LHCB1.2* gene.

Overall, this evidence suggests that overexpression of MORF2 does not result in a significant *gun* phenotype.

### The nuclear transcriptome of white and marbled *gun1* seedlings is significantly affected

**Figure 2:**
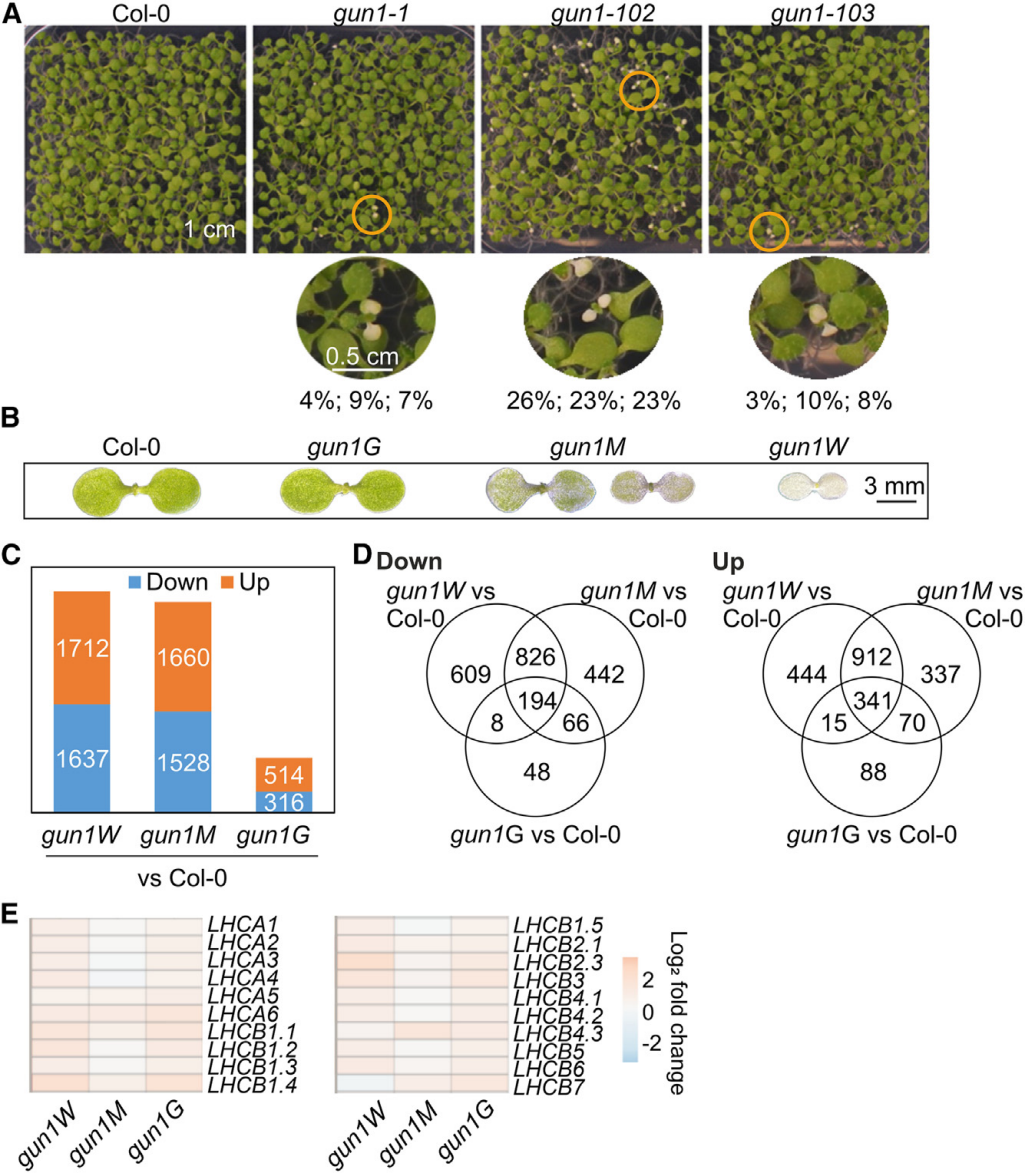
The nuclear transcriptome of white and marbled *gun1* seedlings is significantly affected. **(A)** Phenotypes of 10-day-old Col-0, *gun1-1*, *gun1-102*, and *gun1-103* seedlings grown on MS without inhibitor supplementation under 16-h light/8-h dark conditions. Zoomed-in images were taken of white seedlings, denoted by the circles below the overview pictures. The percentages of abnormal seedlings (white and marbled cotyledons) were calculated for three different seed batches. **(B)** Phenotypes of Col-0, *gun1G*, *gun1M*, and *gun1W* seedlings (derived from *gun1-102*). (**C)** Analysis of transcriptome changes in white (*gun1W*), marbled (*gun1M*), and green (*gun1G*) *gun1-102* mutant seedlings. The numbers represent genes with at least a two-fold reduction (down) or elevation (up) compared with the Col-0 WT control. **(D)** Venn diagrams depicting the degree of overlap between the sets of genes whose expression levels were altered at least two-fold in *gun1W*, *gun1M*, and *gun1G* compared with the Col-0 control. **(E)** Heatmap showing transcript accumulation of genes encoding chlorophyll *a*/*b* binding proteins.

During experiments examining the role of GUN1 in NF-mediated editing changes, we observed the appearance of *gun1* seedlings with white (*gun1W*) and marbled (*gun1M*) cotyledons among the green *gun1* (*gun1G*) seedlings grown on MS medium without inhibitors (Figure 2A). This phenomenon has also been reported previously (Ruckle et al., 2007), but at lower frequencies, which we will discuss later. The phenotype was most pronounced in *gun1-102* seedlings but was also observed in *gun1-1* and *gun1-103* seedlings. The emerging true leaves turned green, suggesting that GUN1 has a specific role in chloroplast development in the cotyledons, consistent with the particular accumulation of GUN1 protein at early stages of cotyledon development (Wu et al., 2018). To obtain a general overview of RNA expression patterns in these prominent *gun1* seedlings, RNA isolated from 4-day-old Col-0 and *gun1W*, -*M*, and -*G* mutant seedlings (Figure 2B) was subjected to lncRNA-seq. Absence of transcription in a portion of exon 2 and subsequent exons of the *GUN1* gene was verified in all *gun1* mutant seedlings (Supplemental Figure 4), confirming the presence of the transfer DNA insertion in all *gun1* seedlings and validating the RNA-seq data. The strong phenotype of *gun1W* seedlings in particular suggests that the post(transcriptome) may be pleiotropically affected. The severity of the *gun1* phenotype was correlated with an increased number of de-regulated genes (Figure 2C). The expression of 3349 genes (including chimeras) changed significantly in *gun1W seedlings compared with* Col-0 (>two-fold, *P* < 0.05; Supplemental Table 1). Among these genes, 1637 showed decreased expression and 1712 showed increased expression, and the numbers of de-regulated genes in *gun1M* and *gun1G* were 3188 and 830, respectively (Figures 2C and 2D). mRNA expression of the marker gene *LHCB1.2* showed only a mild decrease compared with the significant reduction in Col-0 seedlings treated with LIN or NF. This pattern was evident for nearly all of the *LHC* members (Figure 2G).

In summary, the lack of GUN1 in *gun1W* and *gun1M* seedlings has a substantial effect on the nuclear transcriptome, but expression of *LHC* transcripts is only mildly decreased.

### GUN1 deficiency has a significant impact on the entire chloroplast transcriptome

**Figure 3:**
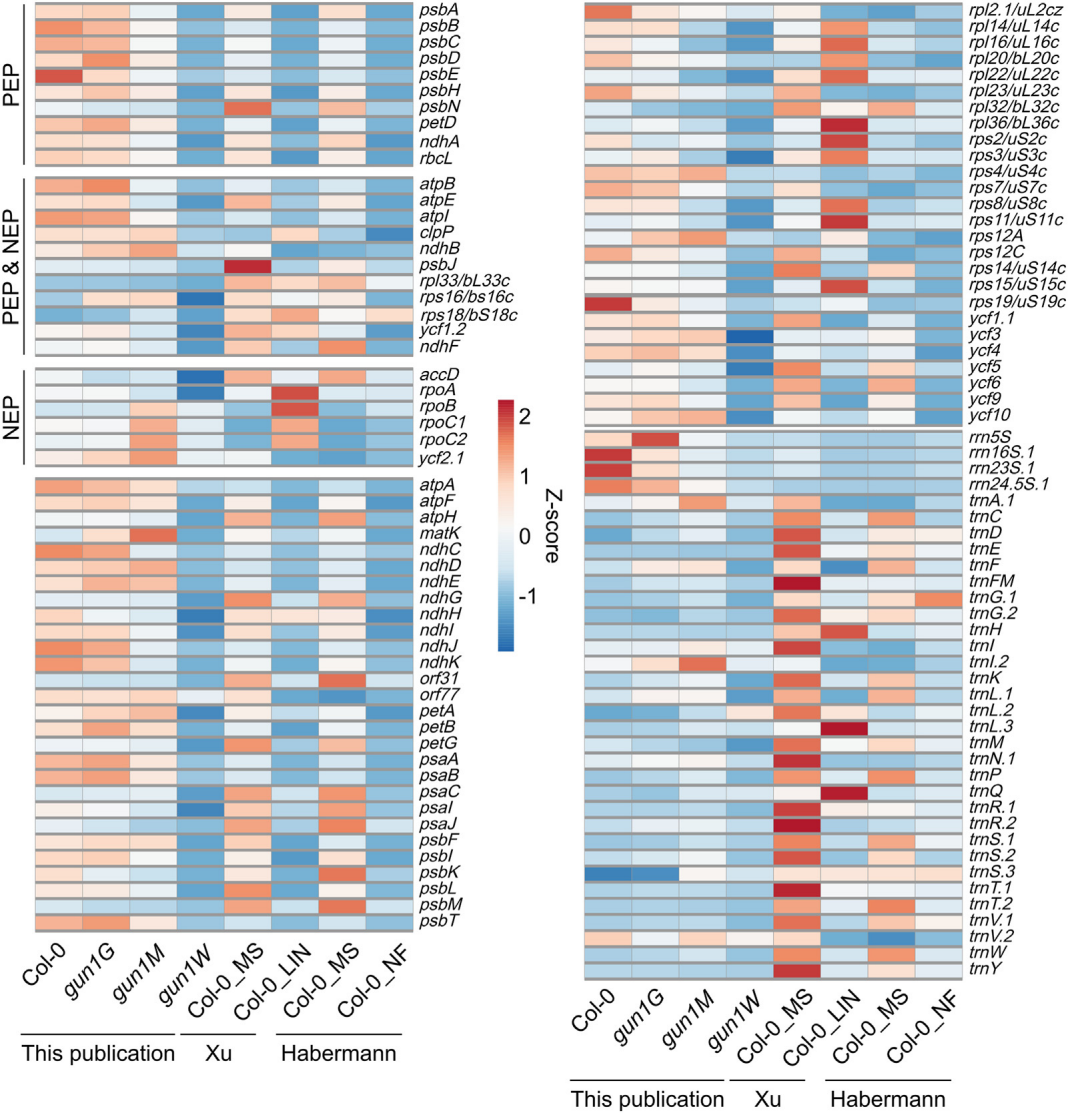
Heatmap illustrating the impact of GUN1 deficiency and NF and LIN treatment on plastid-encoded transcripts (*Z* scores). Low to high expression is represented by the blue to red transition. Note that *Z* scores are calculated for each individual transcript over the different genotypes. NEP is a single-subunit enzyme, whereas PEP consists of core subunits that are encoded by the plastid genes *rpoA*, *rpoB*, *rpoC1*, and *rpoC2* (which are transcribed by NEP) and additional protein factors (sigma factors and polymerase-associated proteins [PAPs]) encoded by the nuclear genome (Borner et al., 2015; Liebers et al., 2018). The general picture has been that only PEP transcribes photosystem I and II genes (*psa* and *psb*), most other genes have both NEP and PEP promoters, and NEP alone transcribes a few housekeeping genes (*rpoB*, *accD*, *ycf2*) (Hajdukiewicz et al., 1997). However, more recent analyses have shown that the division of labor between NEPs and PEPs is more complex (Legen et al., 2002; Borner et al., 2015), and no clear conclusion can be drawn about PEP- or NEP-dependent transcription in *gun1W*: the so-called PEP-dependent genes had lower expression in *gun1W* than in Col-0, as did the genes transcribed by PEP and NEP, although to a lesser extent. NEP-dependent gene expression was also reduced or in the range of Col-0. The transcriptome changes in lincomycin (LIN)-treated (Xu et al., 2020) and NF-treated (Habermann et al., 2020) seedlings were reanalyzed in the same way as the sequencing data generated for this publication. NEP, nuclear-encoded RNA polymerase; PEP, plastid-encoded RNA polymerase.

**Figure 4:**
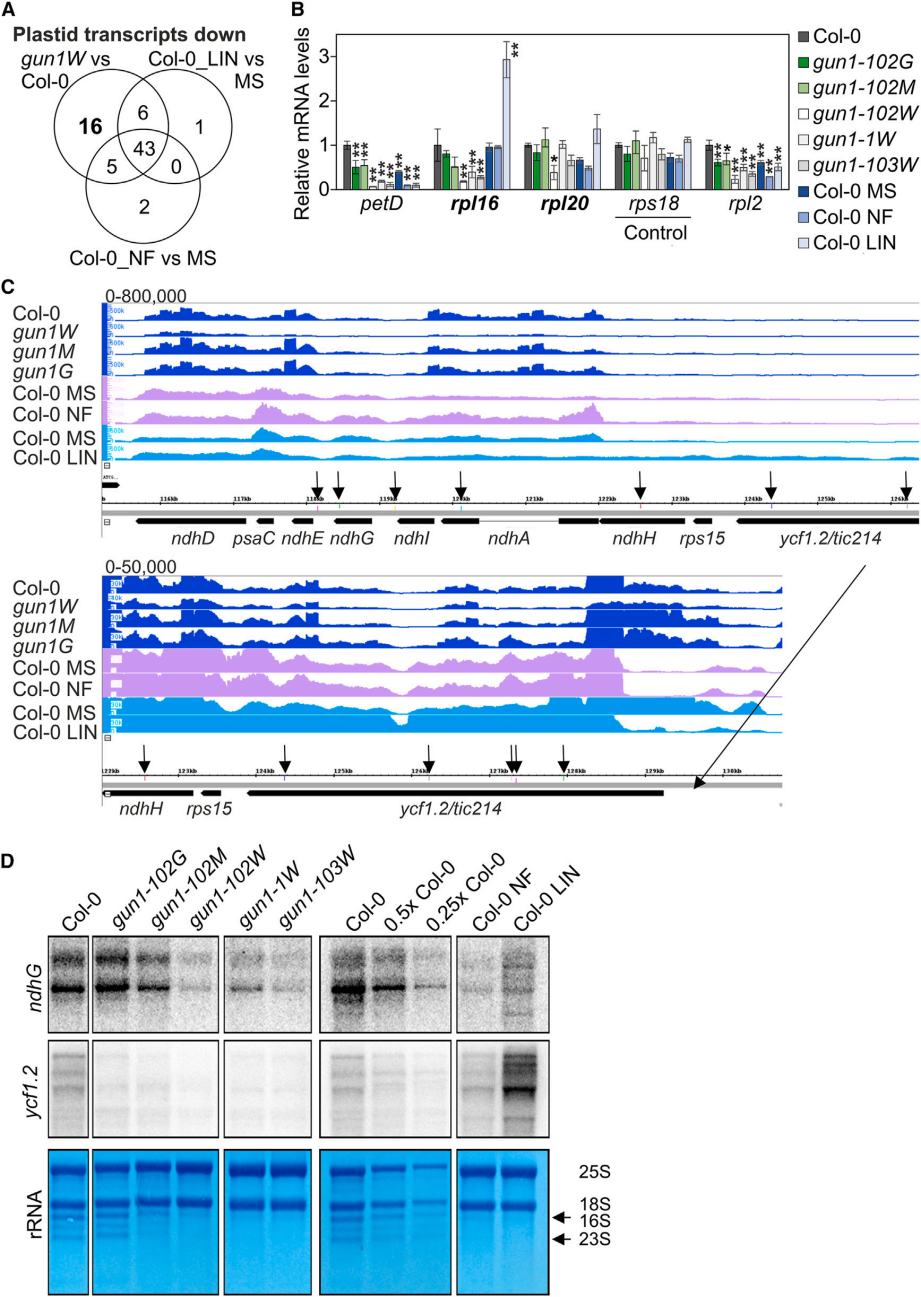
GUN1 deficiency has a significant impact on the chloroplast transcriptome. **(A)** Venn diagrams depicting the degree of overlap between the sets of plastid protein-coding genes whose RNA expression levels were reduced by at least two-fold in *gun1W* relative to Col-0, as well as in LIN- and NF-treated seedlings compared with Col-0 grown on medium without inhibitor (MS). The transcripts of inverted repeat B have been omitted. Note that for the transcripts downregulated by LIN or NF, the adjusted *P* value may also be higher than 0.05. **(B)** RT–qPCR was used to determine expression levels of selected chloroplast transcripts. The results were normalized to the expression of *AT4G36800*, which encodes a RUB1-conjugating enzyme (RCE1). Expression values are reported relative to the corresponding transcript levels in Col-0, which were set to 1. Mean values ± SE were derived from three independent experiments, each performed with three technical replicates per sample. Statistically significant differences (post hoc Tukey’s HSD test; ∗*P* < 0.05 and ∗∗*P* < 0.01) between Col-0 (batch grown together with *gun1* seedlings), *gun1* mutants, and Col-0 seedlings grown on MS, NF, or LIN are indicated by black asterisks. Transcripts marked in bold were downregulated exclusively in *gun1W* but not under NF or LIN treatment. **(C)** Coverage plots depict the accumulation of reads across the *ycf1.2*–*rps15*–*ndhH*–*ndhA*–*ndhI*–*ndhG*–*ndhE*–*psaC*–*ndhD* gene cluster. Vertical arrows point to predicted GUN1 binding sites (see Figure 6; Supplemental Table 6). **(D)** Analysis of *ndhG* and *ycf1.2* transcript accumulation by northern blotting. Total RNA was isolated from 4-day-old Col-0 and *gun1-102* white, marbled, and green seedlings, as well as from Col-0 seedlings grown on medium supplemented with NF or LIN. The samples were run on the same gel but rearranged for clarity. As a loading control and for visualization of rRNAs, the membrane was stained with M.B. The arrows point to bands representing chloroplast rRNAs.

Both NF- and LIN-treated seedlings are bleached to the same degree as *gun1W* seedlings. Therefore, the following analyses involve data previously generated from NF-treated (Habermann et al., 2020) and LIN-treated (Xu et al., 2020) seedlings to account for putative pleiotropic effects in *gun1W* seedlings. Reads from these published data sets were analyzed using the same methodology as that used for our own data (Supplemental Tables 2 and 3). For the plastid transcriptome, we aimed to identify loci for which the relative ratio of editing or splicing was lower in *gun1W*/Col-0, progressively rescued in *gun1M*/Col-0 and *gun1G*/Col-0, and WT-like in NF/MS or LIN/MS. We concluded that the absence of GUN1 does not result in significant changes in chloroplast splicing or editing events (Supplemental Figures 5 and 6). However, plastid transcript levels of 91 out of 133 transcripts (including tRNAs, rRNA, and inverted repeats) were significantly reduced in *gun1W* compared with WT, and no transcripts were significantly induced (Supplemental Table 2). Transcription of chloroplast genes relies on plastid-encoded polymerases (PEPs) and nuclear-encoded polymerases (NEPs) (Borner et al., 2015; Liebers et al., 2018). No clear conclusion can be drawn about PEP- or NEP-dependent transcription in *gun1W*: expression of the so-called PEP-dependent genes was lower in *gun1W* than in Col-0, as was that of the genes transcribed by PEP and NEP, although to a lesser extent. NEP-dependent gene expression was also reduced or in the range of Col-0 (Figure 3; Supplemental Table 2). Note that in the following, our focus is on protein-coding genes, as tRNAs and rRNAs are not reliably detected by the RNA-seq protocol used. When we examined transcript accumulation of protein-coding genes in *gun1W* and NF- and LIN-treated Col-0 seedlings in parallel, we observed, remarkably, that 16 transcripts (excluding transcripts from inverted repeat B) were exclusively decreased in *gun1W* (Figure 4A; Supplemental Figure 7A; Supplemental Tables 2 and 3). This may be due to the use of different growth conditions. Whereas we used 4-day-old seedlings grown under long-day conditions, the NF-treated (Habermann et al., 2020) and LIN-treated (Xu et al., 2020) seedlings were grown under continuous light conditions for 4 and 5 days, respectively. We therefore performed an RT–qPCR experiment using seedlings grown under the same growth conditions (4-day-old seedlings grown under long days) and confirmed the transcript accumulation behavior of *rpl16* and *rpl20* (Figure 4B). Apart from a few genes, most plastid genes belong to polycistronic units and are co-transcribed (Shahar et al., 2019). A closer look at transcripts exclusively reduced in *gun1W*, gradually increased in *gun1M*, and WT-like in *gun1G* drew our attention to a large polycistron containing *rpoA* along with several *rps* and *rpl* genes (Supplemental Figure 7B). Inspection of the coverage plots and transcript accumulation data revealed a comparable behavior for *ycf1.2*, *rps15*, and the *ndhH*–*ndhA*–*ndhI*–*ndhG*–*ndhE*–*psaC*–*ndhD* gene cluster (Figure 4C). The downregulation of transcripts was verified by northern blot detection of *ndhG* and *ycf1.2* (Figure 4D). It is noteworthy that although *ndhG* transcripts did not appear to be reduced in the RNA-seq data of Habermann et al. (2020), the transcript pattern and abundance in *gun1W* plants looked the same as those in Col-0 NF plants under our growth conditions, and therefore, a secondary effect of reduced *ndhG* transcripts in *gun1* seedlings cannot be excluded at this stage. By contrast, *ycf1.2* transcripts appear to be specifically reduced in *gun1W* compared with inhibitor-treated WT. In addition, during the quality control of RNA for sequencing, we observed strong rRNA depletion in *gun1W*, which was gradually rescued in *gun1M* and completely restored in *gun1G* (Figure 4D; Supplemental Figure 8A). The rRNA depletion phenotype was similar to that of Col-0 seedlings treated with NF or LIN (Figure 4D). Therefore, also for this pattern, a secondary effect cannot be excluded at this stage.

In conclusion, the plastid (post)transcriptome is significantly affected by GUN1 deficiency in *gun1W* and *gun1M* seedlings.

### Re-evaluation of a putative RNA-binding function of GUN1

**Figure 5:**
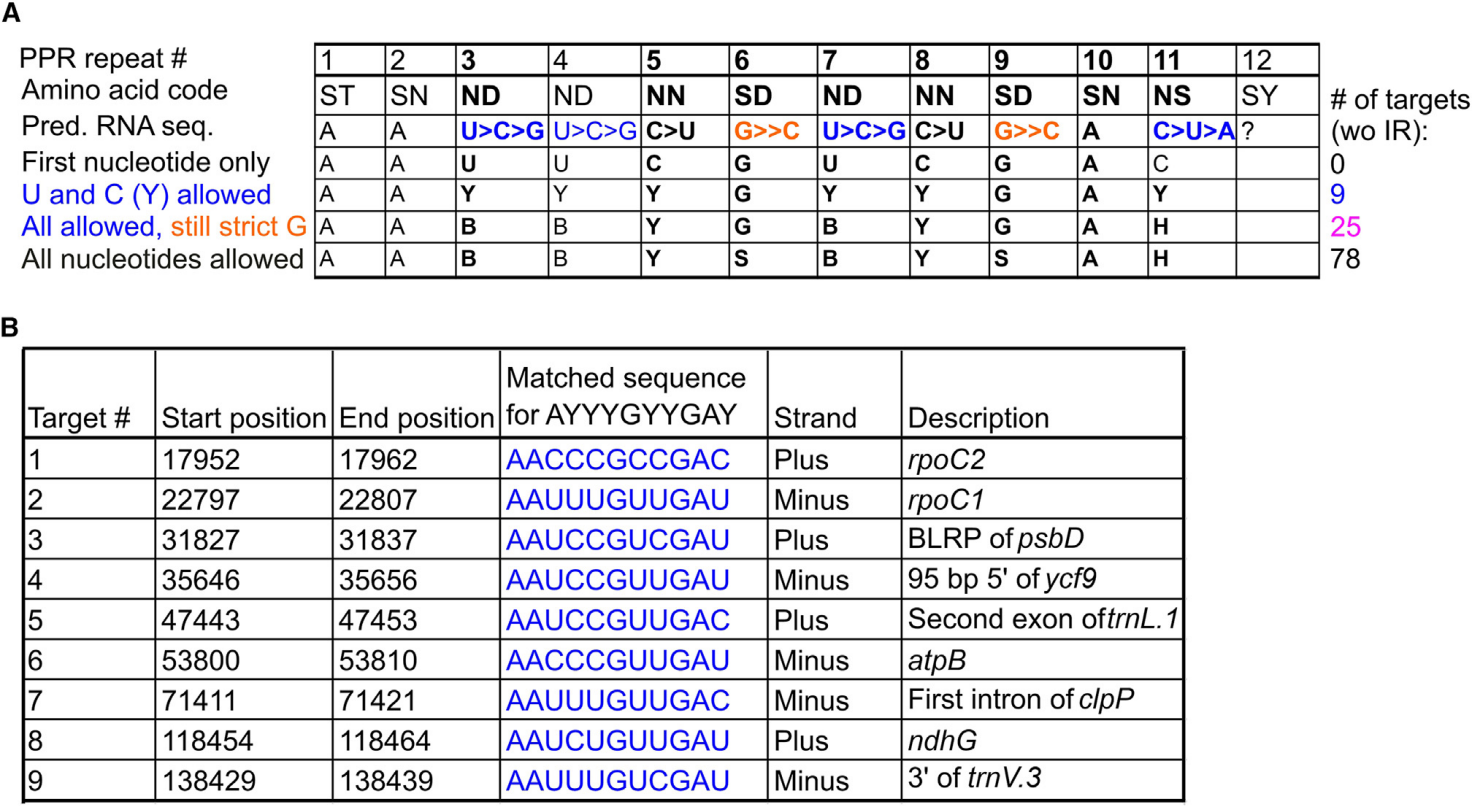
Predicted GUN1 binding sites. **(A)** Predicted ambiguous GUN1 target sequence. The numbers in the first row depict the PPR motif number, whereas the second row displays the amino acids in each PPR motif that are crucial for prediction of target nucleotides. For some amino acid combinations, the predicted target nucleotide is unique (such as ST and SN), whereas for others (such as ND), multiple nucleotides are predicted with descending preference. Subsequent rows indicate the prospective target sequences dependent on the stringency applied to the predicted nucleotides. For example, using only the first nucleotide of each of the predicted nucleotides results in 0 target sites. Allowing U, C, or G for the ambiguous B and G or C for “G>>C” results in 78 potential target sites. Allowing U, C, or G for the ambiguous B and only G for “G>>C” results in 25 potential target sites (here and in the following, marked in magenta). Allowing only U or C for the ambiguous Y and only G for “G>>C” results in 9 potential target sites (here and in the following, marked in blue). Highly conserved regions in GUN1 are highlighted in bold letters, according to Honkanen and Small (2022). In addition, representative predicted binding sites at *ndhG*, *ndhE*, and *rrn23S* are shown. wo IR, without inverted repeat. **(B)** Table showing the nine sites in the “U and C (Y)” category.

Many of the significant changes observed in the chloroplast (post)transcriptomes of *gun1W* and *gun1M* could explain their seedling phenotypes. But what is the primary cause? GUN1 is a P-type PPR protein, suggesting that it may be associated with RNA cleavage, splicing, and stabilization (Barkan and Small, 2014), and this led us to revisit a putative direct RNA-binding function of GUN1. PPR motifs bind to RNA in a one-repeat and one-nucleotide manner, and PPR motifs recognize specific RNA bases through amino acids at positions 5 and 35. Using this code, the binding sites of several PPR proteins can be predicted very well (Shen et al., 2016; Miranda et al., 2018; Yan et al., 2019). Because the correct PPR code is crucial for determining the binding sequence, we investigated the structural configuration of the GUN1 protein by modeling with PyMOL and found that the 12 PPR domains of GUN1 predicted by ScanProsite should be shifted by one amino acid (Supplemental Figure 9). We therefore adjusted the repeat annotation to better fit the predicted structure and description of canonical PPR tracts (Yan et al., 2019; Honkanen and Small, 2022). Prediction of putative RNA target sites (Yan et al., 2019) yielded the following ambiguous 11-nucleotide sequence: 5′-AA(U>C>G)(U>C>G)(C>U)(G>>C)(U>C>G)(C>U)(G>>C)A(C>U>A)-3' (Figure 5A). Using this ambiguous sequence and considering location in inverted repeat regions, 78 potential target sites can be identified within the chloroplast genome, distributed over 41 gene loci (Supplemental Table 4). The application of strict and very strict sequence-matching criteria, as explained in the figure legend to Figure 5A, yields 25 and 9 possible targets, respectively. On the basis of our previous analysis, two regions are noteworthy. One is the *ycf1.2*–*rps15*–*ndhH*–*ndhA*–*ndhI*–*ndhG*–*ndhE*–*psaC*–*ndhD* gene cluster (see Figure 4C), which contains ten potential targets. Among these targets, *ndhE* and 3′*ndhI* are also identified with the strict target sequence and *ndhG* with the very strict target sequence (Figure 5B; Supplemental Table 4). The second region is the *rrn23S* gene (Supplemental Figure 10), which contains four predicted target sequences: 23S_104766, 23S_104856, 23S_106002, and 23S_106558 (numbered according to the nucleotide position in the plastid genome). 23S_104856 and 23S_106558 fall within the strict possible targets. To gain insight into the accumulation of reads across the rRNA operon, we performed lncRNA-seq again without rRNA depletion. This analysis confirmed that plastid rRNAs are significantly reduced in *gun1W* and *gun1M* seedlings (Supplemental Figure 10). Upon closer examination of the first two binding sites and adjustment of the plots for differences in expression, a disproportionately high number of reads were found to map 5′ to the *rrn23S* gene, which is not present in *gun1G* (Supplemental Figure 10). In addition, a distinct coverage pattern of *rrn23S* was observed in the region of binding site 23S_106558, although a secondary effect on 23S rRNA still cannot be excluded.

### GUN1 binds to chloroplast RNAs *in vivo* and *in vitro*

**Figure 6:**
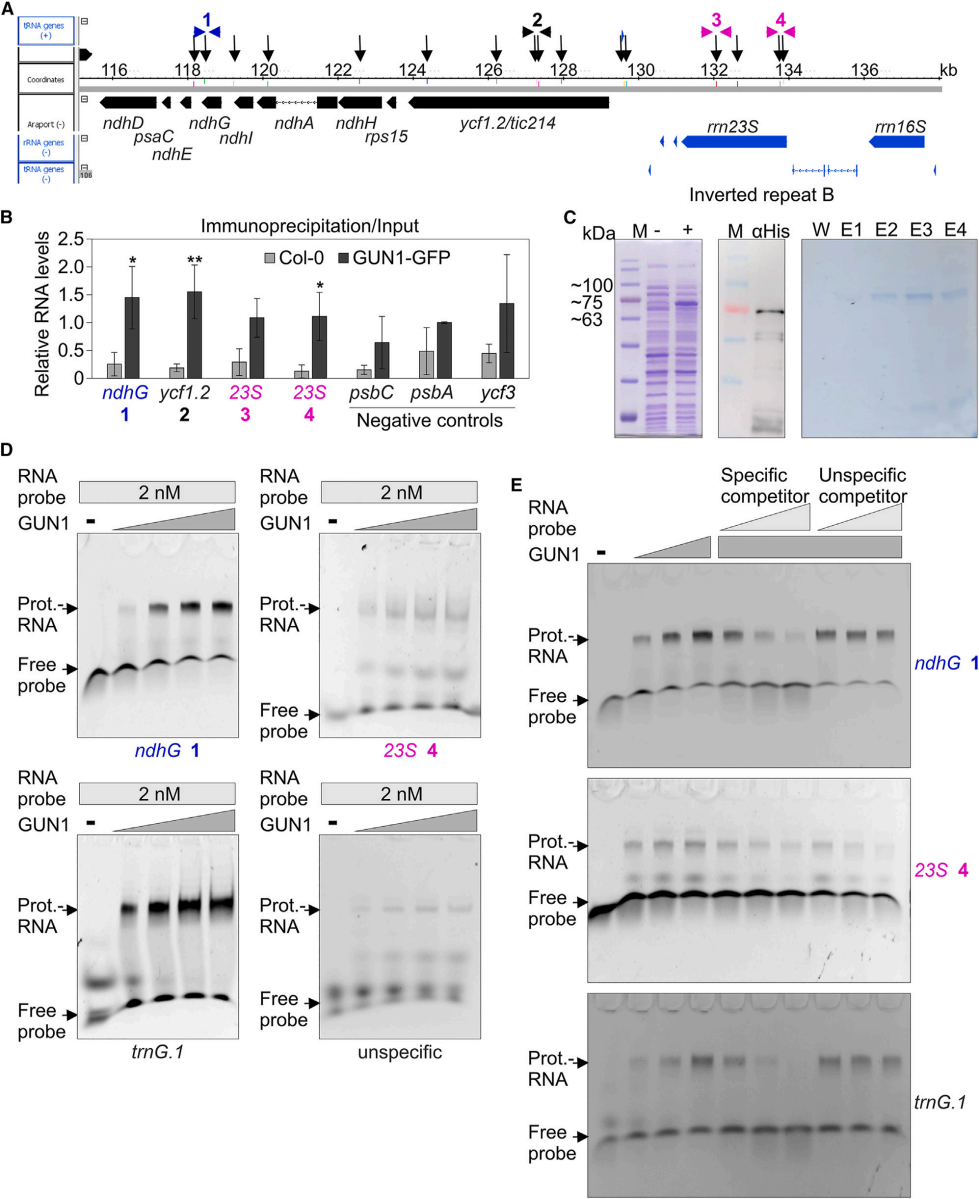
GUN1 binds to RNAs *in vivo* and *in vitro*. **(A**) Schematic presentation of predicted RNA binding sites (indicated by black vertical arrows) in *ycf1.2*, the *rps15*–*ndhH*–*ndhA*–*ndhI*–*ndhG*–*ndhE*–*psaC*–*ndhD* polycistron, and the *rrn23S* gene. Positions of primers used in **(B)** are depicted with arrowheads using the color code explained in the legend to Figure 5. **(B)** Demonstration of co-purification of selected RNAs with GUN1. RNAs that were isolated from the pellet after Co-IP experiments with Col-0 and a GUN1 overexpression line (GUN1–GFP) (IP) and the respective input RNAs (Input) were amplified by RT–qPCR. Ratios of immunoprecipitated versus input RNA levels are reported relative to the corresponding levels in the first Col-0 replicate, which were set to 1. Mean values ± SD were derived from three independent experiments, each performed with three technical replicates per sample. Statistically significant differences (post hoc Tukey’s HSD test; ∗*P* < 0.05 and ∗∗*P* < 0.01) between GUN1–GFP and Col-0 lines are indicated by black asterisks. **(C)** Overexpression and purification of a His-tagged GUN1–PS protein in *E. coli*. GUN1–PS encompasses all PPR and SMR motifs (PS) spanning amino acids 232 to 918. Left: SDS–PAGE before (−) and after (+) 20 h of induction at 18°C; middle: western blot of the induced protein with an anti-His antibody; right: SDS–PAGE after purification. W, wash fraction with a buffer containing 20 mM imidazole; E1 and E2, elution fractions with a buffer containing 250 mM imidazole; E3 and E4, elution fractions with a buffer containing 500 mM imidazole. **(D)** The GUN1 protein interacts *in vitro* with RNA sequences located in *ndhG* and *trnG*. EMSAs were performed with purified His-tagged GUN1 protein that was produced in *E. coli*. Aliquots (0, 100, 200, 400, and 600 nM) of purified GUN1 protein were incubated with Cy5-labeled single-stranded RNA (ssRNA) probes representing the putative target sequences and an nonspecific ssRNA probe. Binding reactions were performed at 23°C, followed by electrophoresis on non-denaturing TBE polyacrylamide gels at 4°C. (**E)** Aliquots (0, 200, and 400 nM) of purified GUN1 protein were incubated with Cy5-labeled ssRNA probes in the presence of increasing concentrations (5×, 25×, 50×; indicated by the light gray triangle) of the same unlabeled ssRNA (specific) or a nonlabeled ssRNA of unrelated sequence (nonspecific) as competitors. Binding reactions were then subjected to electrophoresis on non-denaturing TBE-polyacrylamide gels as performed in **(D)**.

To investigate whether GUN1 is involved in RNA binding *in vivo*, RNA Co-IP was performed using a GPF-tagged GUN1 line (*GUN1–GFP*) (Tadini et al., 2016) with Col-0 as a control. The success of the IP experiment was demonstrated by detection of the tagged proteins in the respective eluates by western blotting (Supplemental Figure 11). Four predicted target regions of the notable regions described above (*ndhG*, *ycf1.2*, and two regions of *23S* rRNA; Figure 6A) along with negative controls were tested in RT–qPCRs of input and immunoprecipitated RNA, and the input/immunoprecipitated ratio was calculated. In GUN1 IPs, *ndhG*, *ycf1.2*, and a target in *23S* rRNA comprising binding sites 104766 and 104856 demonstrated significant enrichment in the pellet compared with the control (Figure 6B). By contrast, there was no significant enrichment of RNAs that lacked predicted target sites. Also, binding of GUN1 to *23S*_106558 was not statistically significant. However, the identification of *ndhG*, *ycf1.2*, and *23S* rRNA as true targets must be considered with caution. First, all RNAs tested gave a stronger signal in the GUN1 IP than in the control. Second, all negative controls contained a sample with extremely large error bars.

To determine whether GUN1 can directly bind to the identified target sites, we used electrophoretic mobility shift assays (EMSAs). It is difficult to obtain full-length GUN1 by overexpression in *E. coli*, possibly owing to the highly disordered domain in the N-terminal region (Shimizu et al., 2019). Therefore, we overexpressed a GUN1–PS construct encompassing all PPR and SMR motifs (PS) spanning amino acids 232 to 918 (Shimizu et al., 2019) in *E. coli* (Figure 6C) and used GUN1–PS for EMSAs. Four different Cy5-labeled RNA oligonucleotides were designed, representing the putative binding sites at *ndhG*, *23S*_104856, and *trnG.1* and an unrelated sequence. All probes were 25 bp long. The secondary structure of the non-specific probe was represented by a hairpin loop similar in structure to the *ndhG* probe, whereas the *trnG.1* probe formed a more stable hairpin loop, and the *23S* probe formed a predominantly circular loop. When 100, 200, 400, and 600 nM of purified GUN1–PS protein was added to the Cy5-labeled probes and the mixtures electrophoresed, band shifts were observed, especially for the *ndhG* and *trnG.1* probes. The shift was more pronounced at a higher protein concentration and was not detected when no protein or probe was added, indicating that the RNA probes formed complexes with the protein (Figure 6D). A slight shift could also be detected for *23S_104856*. However, the non-specific probe produced a similar shift pattern.

The intensity of the shifted *ndhG* and *trnG.1* bands progressively decreased upon addition of increasing concentrations of the respective unlabeled single-stranded RNAs but not upon addition of increasing concentrations of unlabeled, unrelated single-stranded RNA (Figure 6E). However, the intensity of the *23S* shift decreased upon addition of both the specific and the nonspecific competitor. This suggests that GUN1 binds specifically to the *ndhG* and *trnG.1* target sites but does not bind, or does so only weakly or nonspecifically, to *23S*_*104856*.

**Figure 7:**
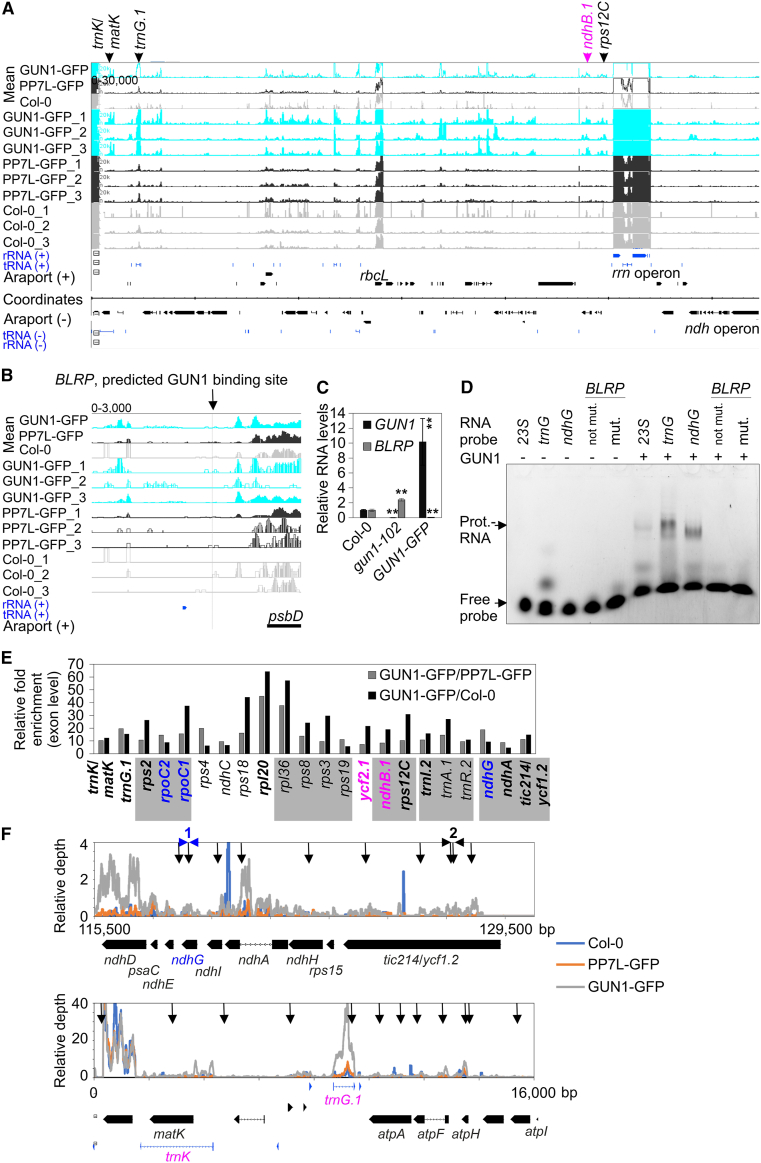
Identification of putative GUN1 targets by RIP-seq analysis. **(A**) Libraries were prepared from RNAs co-immunoprecipitated from a GPF-tagged GUN1 line (GUN1–GFP) and, as controls, from a PP7L–GFP line and Col-0 and then sequenced. The experiment was performed with three biological replicates. Coverage plots of reads per kilobase per million (RPKM) values show the accumulation of reads across the chloroplast genome, here shown without inverted repeat B. Vertical arrows indicate examples of regions with higher read accumulation in GUN1–GFP compared with PP7L–GFP and Col-0 and that also contain a match to the predicted GUN1 target code (see Supplemental Tables 4 and 6). The color code is explained in the legend to Figure 5. **(B**) Coverage plot of RPKM values across the blue light responsive promoter (*BLRP*) of *psbD* encompassing the predicted GUN1 binding site. **(C)** RT–qPCR to determine expression levels of *GUN1* and the *BLRP* region covering the predicted GUN1 binding site. Seedlings were grown under continuous light (100 μmol m^−2^ s^−1^) for 5 days. The results were normalized to *AT4G36800*, which encodes a RUB1-conjugating enzyme (RCE1). Expression values are reported relative to the corresponding transcript levels in Col-0, which were set to 1. Mean values ± SE were derived from three independent experiments, each performed with three technical replicates per sample. Statistically significant differences (post hoc Tukey’s HSD test; ∗*P* < 0.05 and ∗∗*P* < 0.01) between Col-0 and the transgenic lines are shown. **(D)** Under our conditions, the GUN1 protein does not interact *in vitro* with the predicted GUN1 binding site located in the *BLRP*. EMSAs were performed with purified His-tagged GUN1 protein that was produced in *E. coli*. Aliquots (0 and 800 nM) of purified GUN1 protein were incubated with 2 nM Cy5-labeled ssRNA probes representing the putative target sequences and a *BLRP* probe containing 10 mutated sites (mut.). Binding reactions were performed at 23°C, followed by electrophoresis on non-denaturing TBE polyacrylamide gels at 4°C. **(E)** Libraries were prepared from RNAs isolated from the Co-IP experiments described in **(A)**. Relative enrichment ratios (calculated at the exon level) of GUN1–GFP relative to Col-0 and GUN1–GFP relative to PP7L–GFP are shown. Gray shading indicates genes located in a polycistron. Transcripts that also contain a match to the predicted GUN1 target code (see Supplemental Tables 4 and 6) are written in bold. The color code is explained in the legend to Figure 5. **(F)** Plot of RIP-seq data over two example regions. Relative depth was calculated at each nucleotide (nt) position by relating the number of reads to the total depth of the sequencing output. Black vertical arrows indicate predicted GUN1 RNA-binding sites.

To obtain a broader view of the RNA targets of GUN1, libraries prepared from immunoprecipitated RNAs of the GUN1–GFP line and Col-0 were subjected to RNA-seq (RIP-seq). In addition to Col-0, another unrelated GFP-tagged line (PP7L–GFP; Xu et al., 2019) served as a control. Supplemental Table 5 shows the normalized read depths at each position in the chloroplast genome. Coverage files were generated using the bamCoverage tool, set to reads per kilobase per million, and reads were plotted across the entire chloroplast genome. This procedure revealed several read peaks in the GUN1 libraries that were not observed as strongly in plots of the control libraries (Figure 7A). These included, for example, *trnK*/*matK*, *trnG.1*, and *ndhB.1*. One predicted GUN1 target is the blue light responsive promoter (*BLRP*) of *psbD* (see Supplemental Table 4); *psbD* transcript levels are reduced to 11% in *gun1W* relative to the WT (see Supplemental Table 2), and RIP–qPCR and EMSA experiments recently suggested that GUN1 binds to the *BLRP* (Cui et al., 2023). However, our RIP-seq analysis did not show any enrichment of reads at the *BLRP* (Figure 7B), perhaps due to different growth conditions. We used 4-day-old seedlings grown under long-day conditions, whereas Cui et al. (2023) used seedlings grown in the dark for 2.5 days, which were then transferred to light (100 μmol m^−2^ s^−1^) for 6 h. Furthermore, it has been shown that *BLRP* transcripts are strongly reduced in *gun1 p35S*::*GUN1–GFP* seedlings under the above-mentioned light-transfer growth conditions or in 5-day-old seedlings grown under continuous light (Cui et al., 2023), a result that we confirmed for the latter growth condition (Figure 7C). Therefore, failure to detect a peak in the *BLRP* region in our RIP-seq data may be due to insufficient levels of *BLRP* transcript input. To further investigate GUN1 binding to the *BLRP* region, we performed EMSA experiments with our *23S_104856*, *trnG.1*, and *ndhG* probes and the RNA1 and RNA3 probes designed by Cui et al. (2023). RNA1 includes the *BLRP* GUN1 binding site, and RNA3 is a probe with 10 mutation sites in the *BLRP* binding region. Addition of 800 nM purified GUN1 protein to 2 nM of each probe resulted in shifts of the *trnG.1* and *ndhG* probes, a weaker shift of the *23S_104856* probe*,* and a faint shifted smear of the RNA1 and RNA3 probes (Figure 7D). Two additional independent experiments produced similar results (Supplemental Figure 12A). Because we performed the binding reactions at 23°C and ran them at 4°C, we repeated the EMSA experiments twice using the conditions of Cui et al. (2023), who performed the binding reactions at 37°C and ran them at room temperature, again showing similar results (Supplemental Figure 12B). There was no clear shift of the RNA1 probe, and the binding reaction with the RNA3 probe—which contained the mutation sites—behaved similarly to that with RNA1, although a shift was visible for *trnG.1* and *ndhG*. Therefore, under our RIP-seq and EMSA conditions with our GUN1–PS protein, we did not observe a shift of the *BLRP* GUN1 target. It should be noted that our GUN1–PS comprises amino acids 232 to 918, whereas the GUN1 protein expressed by Cui et al. (2023) contained 100 additional amino acids: it encompassed amino acids 132 to 918, and we cannot exclude the possibility that these 100 additional amino acids are required for *BLRP* binding.

Enrichment analysis at the exon level compared with RNAs identified in the control lines showed that 22 transcripts were significantly enriched in GUN1–GFP (Figure 7E; Supplemental Table 6); 13 of them contained at least one predicted GUN1 target region, covering a total of 26 predicted targets. This was a significant enrichment according to three different statistical tests, the chi-squared (*P* = 0.019), hypergeometric (*P* = 0.019), and binomial (*P* = 0.021) tests. The enriched transcripts harboring a predicted GUN1 target site included *ycf1.2*, *ycf2*, *rps2*, *rps12C* and *rpl20*, *rpoC1* and *rpoC2*, *ndhB*, the *ndhH*–*ndhA*–*ndhI*–*ndhG*–*ndhE*–*psaC*–*ndhD* gene cluster, and tRNAs such as *trnK*, *trnG.1*, and *trnI.2* (Figure 7E and 7F).

It is important to note that we did not sequence input libraries, and we only confirmed significant IP/input ratios for three targets (see Figure 6B). Overall, however, these experiments provide evidence for an RNA-binding function of GUN1 and suggest candidates for further testing.

## Discussion

Although the functions of other GUN proteins are well established, the specific molecular function of GUN1 has remained largely unclear. Most conclusions regarding GUN1 have been made by examining *gun1* mutants in combination with inhibitor treatments or in conjunction with the generation of double mutants (Richter et al., 2023). Our observation of *gun1W* and *gun1M* seedlings is independent of NF or LIN treatment. In these seedlings, the emerging true leaves turned green, suggesting a specific role for GUN1 in chloroplast development in cotyledons. This is consistent with the particular accumulation of GUN1 at early stages of cotyledon development (Wu et al., 2018).

### “Same genotype, different phenotype” phenomenon

The prevailing view of *gun1* mutants is that adult plants exhibit no noteworthy phenotypes under normal growth conditions, apart from earlier flowering (Wu et al., 2018; Marino et al., 2019). Most inferences related to GUN1 were made when the *gun1* mutant was examined under stressful conditions, in combination with inhibitor treatments, or in conjunction with the creation of double mutants (see the introduction). We observed the appearance of *gun1* seedlings with white (*gun1W*) or marbled (*gun1M*) cotyledons when plants were grown under normal growth conditions and without inhibitor supplementation (see Figure 2). Previous reports also noted the sporadic presence of variegated (observed in *gun1-1* and *gun1-101*; Ruckle et al., 2007) or paler (observed in *gun1-101*; Wu et al., 2018) cotyledons. It is interesting to note that seedlings with the same genotype can exhibit various phenotypes. This phenomenon, described as incomplete penetrance and variable expressivity, is widely discussed in the animal field because of its relevance for diseases (Kingdom and Wright, 2022). Epigenetic modifications and environmental effects are potential factors that could contribute to this phenomenon. Environmental effects on mutants impaired in PGE have been observed, as in the case of *gun1* mutants, which exhibit a defect in cold acclimation (Marino et al., 2019). However, we can exclude a purely environmental cause for the appearance of *gun1W* seedlings, as they were interspersed among green *gun1* seedlings on the same plate. Epigenetic changes, specifically DNA methylation and histone modifications, can affect gene expression without modifying the DNA sequence. Again, these changes can be influenced by environmental factors and can result in distinct phenotypes despite identical genotypes. The *gun1W* seedlings were observed in diverse laboratories with different generations and *gun1* alleles, including complete knockouts (*gun1-101* and *gun1-102*). Therefore, epigenetics is also unlikely to be the primary/sole contributing factor. A comparable scenario to that of *gun1* mutants was described for the *immutants* and *variegated2* mutants. Nevertheless, these mutants exhibited green and white sectors within the same leaf. Discussion of these mutants revolves around the compensatory mechanisms and the concept of plastid autonomy for both mutants. However, although redundant gene products are suggested to be involved in *variegated2*, they are not implicated in *immutants*. The hypothesis is that the attainment of certain activity thresholds is required for the proper development of chloroplasts (Yu et al., 2007), and this may also apply for the *gun1* mutants. A threshold effect would also explain the sensitivity of *gun1* mutants to LIN, NF (Song et al., 2018; Zhao et al., 2018), and abscisic acid (Cottage et al., 2010) during early seedling development. Recently, a *gun1* molecular phenotype was identified under non-stressful conditions. This phenotype included lower activities of both superoxide dismutase and ascorbate peroxidase and, consequently, higher superoxide anion concentrations and lipid peroxidation compared with the WT, suggesting that GUN1 may protect chloroplasts from oxidative damage (Fortunato et al., 2022). The phenotype could also be influenced by the presence of modifier genes that can suppress or enhance the mutant phenotype, as observed for floral trait variation, which is highly dependent on ecotype (Juenger et al., 2000). In the absence of GUN1, compensatory mechanisms may be activated during seedling development, and the failure of compensation in only a subset of the population is likely dependent on the intensity or specific nature of environmental stresses experienced by the parent plants. This, in turn, may indicate that there are critical thresholds of environmental factors beyond which the compensation is inadequate, leading to phenotypic variability within the population. In addition, GUN1 protein accumulates at the early stages of cotyledon development, and the timing of gene expression during development is known to influence penetrance (Kingdom and Wright, 2022). However, further analysis is needed and may include how stochastic factors—such as segregation of organelle genomes through development and reproduction (Broz et al., 2024)—in conjunction with environmental factors and transgenerational effects contribute to the development of individual phenotypes (Burga and Lehner, 2012).

### Functions of GUN1 in plastid transcript maturation

GUN1 was previously suggested to regulate plastid RNA editing during NF treatment of seedlings (Zhao et al., 2019). The proposed mechanism involved the interaction of GUN1 with MORF2 and did not require the direct interaction of GUN1 with the target transcript, which was a logical explanation because no *in vivo* RNA-binding function of GUN1 had been demonstrated to date. However, the role of GUN1 in editing and its contribution to GUN signaling have not yet been satisfactorily resolved for several reasons. First, the oeMORF2 *gun* phenotype has been postulated for NF treatment but not LIN treatment. Second, the slight differences in editing performance between Col-0 and *gun1* during NF treatment (see Figure 1) are unlikely to be the trigger for retrograde signaling. Third, editing of relevant sites was more or equally suppressed in oeMORF2 compared with *gun1-9*. One would therefore expect oeMORF2 lines to be even stronger *gun* mutants than *gun1* itself, but this was not the case for both our data and data generated by Zhao et al. (2019) (see Figure 1). Here, it should be noted that our oeMORF2 lines had lower *MORF2* mRNA expression levels than those generated by Zhao et al. (2019). However, other studies have also not found any involvement of GUN1 in editing changes in other retrograde signaling processes (Kakizaki et al., 2012; Loudya et al., 2020). Furthermore, GUN1 is classified as a member of the P-type PPR proteins, which rarely have a direct role in editing (Small et al., 2020).

GUN1 is one of the five PPR-SMR chloroplast-located proteins, all of which have essential functions in chloroplast development (see Figure 2; Zhang and Lu, 2019). Interestingly, GUN1 protein is present at very low levels and is barely detectable by proteomic approaches, whereas the other PPR-SMR proteins are particularly abundant compared with most PPR proteins (Liu et al., 2013). This fact, together with the distinct (post)transcriptome of *gun1* mutants (see Figure 3), may be important for the unique function of GUN1 in GUN signaling, as *svr7* and *sot1* mutants are not *gun* mutants (Wu et al., 2016). Interestingly, plastid rRNA accumulation is impaired in mutants of the three proteins SVR7, SOT1, and GUN1. Whereas SOT1 (Wu et al., 2016; Zhou et al., 2017) binds directly to the (precursor) *23S* rRNA, this is not clear for SVR7 and is questionable for GUN1 (see Figure 6). Therefore, the defect in rRNA accumulation in the *svr7* mutant and *gun1W* and *gun1M* seedlings may be a secondary effect. However, the primary function of SVR7 is to ensure correct expression of the ATP synthase (Zoschke et al., 2013). For SOT1, specifically its function in rRNA maturation has been investigated, and it has been shown that the SMR domain has endonuclease activity (Wu et al., 2016; Zhou et al., 2017), but other targets are, to date, unknown. Interestingly, in contrast to those in the *gun1* mutant, the plastid transcripts of protein-coding genes (except *ndhA*) in *sot1* tend to be slightly upregulated (Yan et al., 2019), whereas the *gun1* (post)transcriptome is greatly affected, and we identified a plethora of enriched RNA sites in our RIP-seq experiment (see Figure 7).

The significantly reduced plastid rRNA levels (Scharff and Bock, 2014) would be sufficient to explain the *gun1W* phenotype, although this reduction is likely to be a secondary effect. The determination of theoretical targets of GUN1 on the basis of its PPR code and enriched targets by RIP-seq analysis suggests that *ycf1.2*, *ycf2*, *rps2*, *rps12C* and *rpl20*, *rpoC1* and *rpoC2*, *ndhB*, *ndhA* and *ndhG*, *matK*, and tRNAs such as *trnK*, *trnG.1*, and *trnI.2* are putative targets. Moreover, EMSA analysis suggests *in vitro* binding of GUN1 to two of these targets, *ndhG* and *trnG.1*. However, whether *ndhG* and *trnG.1* are authentic physiological targets of GUN1 still remains to be determined. NdhG is a component of the NAD(P)H dehydrogenase (NDH) complex. As discussed above, GUN1 is needed for cold acclimation (Marino et al., 2019), and GUN1 may protect chloroplasts from oxidative damage (Fortunato et al., 2022). This protection may be achieved by stabilization of the NDH complex to ensure chloroplast function, especially under oxidative stress conditions. However, here it has to be noted that the role of the NDH complex under different stress conditions remains controversial (Yamori and Shikanai, 2016). The *Arabidopsis* plastid genome contains two genes encoding precursor tRNAs specific for glycine: *trnG.1* for tRNA-Gly(UCC) and *trnG.2* for tRNA-Gly(GCC). Through Watson–Crick base pairing and by wobbling, tRNA-Gly(UCC) recognizes GGA and GGG codons, and tRNA-Gly(GCC) reads GGC and GGU triplets (Tiller and Bock, 2014). However, knockout of *trnG.2* in the tobacco plastid genome demonstrated that translation is maintained to some extent, but the trnG-UCC gene encoding tRNA-Gly(UCC) is essential. This suggests that tRNA-Gly(UCC), encoded by *trnG.1*, is sufficient to read all four glycine triplets (Rogalski et al., 2008). The *gun1W* phenotype is not lethal; therefore, an additional protein may be involved in *trnG.1* maturation, or the *gun1-102* mutant may still permit residual GUN1 expression. However, reduced maturation of *trnG.1* and possibly the predicted targets *trnK*, *trnI.2*, *rps2*, *rps12C*, and *rpl20* (all of which are essential) likely results in reduced protein translation, including that of chloroplast-encoded RNA polymerase subunits. This, or a direct effect of GUN1 on *rpoC1* and *rpoC2*, which contain predicted GUN1 target sites, may cause the widespread downregulation of chloroplast transcripts in *gun1W* seedlings.

Interestingly, GUN1 is predicted to bind to multiple sites in *ycf1.2*, and no *ycf1.2* maturation factors have been identified to date. Our data do not reveal precisely how GUN1 performs its function on plastid RNA, which may involve transcript stabilization or endonucleolytic cleavage through its SMR domain. In addition, we do not address how the molecular function of GUN1 relates to retrograde signaling. Nevertheless, we provide strong evidence that GUN1 binds to RNA and suggest target sites. We anticipate that our findings will serve as a foundation for subsequent studies exploring the role of GUN1 in plastid RNA metabolism and retrograde signaling.

## Methods

### Plant material and growth conditions

The *gun1-1* mutant and the transfer DNA insertional mutants *gun1-102* (*SAIL_290_D09*) and *gun1-103* (*SAIL_742_A11*) are derived from the Col-0 ecotype and have been described previously (for example, Shimizu et al., 2019).

To detect editing levels via RT–PCR, surface-sterilized seeds were sown on MS plates containing 0.8% (m/v) agar. The seeds were then stratified for 4 days in the dark at 4°C. Seedlings were grown for 5 days at 22°C under continuous illumination (100 μmol photons m^−2^ s^−1^) provided by white fluorescent lamps. For NF treatment, MS medium was supplemented with or without a 5 μM final concentration of NF (Sigma-Aldrich, 34364).

For RNA-seq and RIP experiments, surface-sterilized seeds were sown on half-strength MS plates containing 1% sucrose. The plates were then kept in the dark at 4°C for 2 days. Following stratification, the seedlings were grown under a 16-h light/8-h dark photoperiod at 22°C with a light intensity of 100 μmol photons m^−2^ s^−1^ for 4 days. For the results shown in Figure 7C, seedlings were grown under continuous light (100 μmol m^−2^ s^−1^) for 5 days after stratification.

### Generation of oeMORF2 transgenic lines

The *35S:MORF2-YFP* transgene was constructed into the pFGC5941 binary transformation vector as described previously (Yapa et al., 2023). To avoid post-transcriptional co-suppression and to stabilize high expression of *MORF2-YFP*, *35S:MORF2-YFP* was transformed into the post-transcriptional gene silencing mutant *sgs3-1* (Butaye et al., 2004). Plants containing a single insertion of *35S:MORF2-YFP* were identified on the basis of a 3:1 (resistant/sensitive) segregation ratio of T2 plants grown on 1/2 MS medium containing 15 mg/L phosphinothricin. Homozygous transgenic plants were obtained in the T3 generation and further self-fertilized to generate T4 plants that were used for phenotypic analysis.

### RNA preparation, cDNA synthesis, and RT–qPCR

Plant material (70 mg) was frozen in liquid nitrogen and then crushed using a TissueLyser (Retsch, model MM400). One milliliter of TRIZOL (Invitrogen, Carlsbad, CA, USA) and 200 μl of chloroform were used for RNA isolation according to the manufacturer’s instructions. RNA was then precipitated from the aqueous phase using isopropyl alcohol, and the resulting RNA pellet was washed with 70% (v/v) ethanol and dissolved in RNase-free water. After DNase I treatment (New England Biolabs [NEB], Ipswich, MA, USA), 10 μg of RNA was further cleaned with the RNA Clean & Concentrator-5 Kit (Zymo Research, Irvine, CA, USA; R1016). The purified RNA (500 ng) was used to synthesize cDNA with the iScript cDNA Synthesis Kit (Bio-Rad). RT–qPCR analysis was performed on a Bio-Rad iQ5 real-time PCR instrument with iQ SYBR Green Supermix (Bio-Rad). The primers used for this assay are listed in Supplemental Table 7. Tukey’s honestly significant difference test was performed using the following website: https://astatsa.com/OneWay_Anova_with_TukeyHSD/.

### RNA editing analysis by amplicon sequencing

The same growth conditions used by Zhao et al. (2019) were applied. Total RNA was isolated from agar-plate-grown seedlings by acid guanidinium thiocyanate–phenol–chloroform-based extraction and purified from the aqueous phase using the Monarch RNA Clean Up Kit (NEB). Genomic DNA in the samples was removed using TURBO DNase (Thermo Fisher Scientific, Waltham, MA, USA), followed by purification with the Monarch RNA Clean Up Kit (NEB). RNA (1 μg per sample) was transcribed to cDNA with Protoscript II reverse transcriptase (NEB). *clpP*, *psbZ*, *rpoC1*, *rpoB*, *ndhB*, and *ndhF* amplicons were amplified from all samples with Q5 polymerase (NEB). Amplification specificity was assessed by agarose gel electrophoresis, and amplicons were then purified with the Monarch PCR & DNA Clean Up Kit (NEB). Resulting DNA concentrations were measured spectrophotometrically with a NanoDrop instrument. Equimolar amounts of all amplicons from a given sample were pooled and analyzed by the Amplicon-EZ service from Genewiz. The resulting 250-bp paired-end reads were mapped with the short-read aligner BBMap (https://sourceforge.net/projects/bbmap) to an amplicon-specific reference. RNA editing was assessed from the mapped reads as described previously (Royan et al., 2021).

### RNA gel-blot analysis

Total RNA was isolated using TRIzol reagent (Thermo Fisher Scientific). RNA samples were digested with DNase I (NEB) to remove genomic DNA. Then, 2 μg of total RNA was electrophoresed on a denaturing formaldehyde gel, transferred to a nylon membrane (Hybond-XL; GE Healthcare, Freiburg, Germany), and cross-linked with UV light. Hybridizations were performed at 65°C overnight according to standard protocols. The results were visualized using the Typhoon scanner (GE Healthcare).

### RNA editing and splicing analysis of lncRNA-seq data

To ascertain the presence of edited and spliced transcripts from organelles from lncRNA-seq datasets, the Chloro-Seq pipeline (Malbert et al., 2018) was used with the modifications described in Xu et al. (2023).

### RNA-seq and data analysis

Total RNA from plants was isolated with Trizol (Invitrogen), purified with Direct-zol RNA MiniPrep Plus columns (Zymo Research), and sequenced as described previously (Xu et al., 2019). RNA-seq reads were analyzed on the Galaxy platform (Afgan et al., 2016) essentially as described previously (Xu et al., 2019) except that reads were first mapped with the gapped-read mapper RNA STAR (Dobin et al., 2013) to generate the coverage plots in a subsequent step. The BAM files generated by RNA STAR were also used to determine the expression levels of chloroplast-encoded genes. To this end, reads were counted with featureCounts (Liao et al., 2014) using the gene annotation in Araport11 (https://www.arabidopsis.org/download/list?dir=Public_Data_Releases%2FTAIR_Data_20230630), allowing multimapping of reads to account for the inverted repeat regions. Differentially expressed genes were identified using DESeq2 (Love et al., 2014) with the fit type set to “parametric,” a linear two-fold change cutoff, and an adjusted *P* < 0.05. To determine expression levels of nuclear-encoded genes, the reads were mapped with Salmon (Patro et al., 2017) to identify differentially expressed genes as described in Xu et al. (2023), except that the updated AtRTD3-QUASI high-resolution transcriptome (Zhang et al., 2022) was used as the reference transcriptome.

### Protein expression and EMSAs

The pET48 AtGUN1–PS plasmid, encoding amino acids 232 to 918 of GUN1 with an N-terminal TRX-His tag, which was published in Shimizu et al. (2019), was obtained from Addgene (plasmid #136358). The plasmid was isolated and then transformed into BL21(DE3) cells (Thermo Fisher Scientific; EC0114) for protein expression. A positive colony was inoculated into Luria-Bertani medium containing 50 μg/ml ampicillin and grown overnight. The overnight culture was then diluted 1:100 and grown to an optical density 600 of 0.5. After cooling on ice for 30 min, 1 M IPTG was added to a final concentration of 1 mM to induce protein expression, and the culture was incubated at 18°C for 20 h. After harvest of bacterial cells by centrifugation at 4°C, the soluble tagged GUN1 protein was extracted and purified using Protino Ni-NTA agarose (Macherey-Nagel, Düren, Germany; #7450400-500) according to the manufacturer’s instructions. Although the pET48 AtGUN1-PS construct tends to form inclusion bodies, purification was attempted from the supernatant to preserve the native state of the GUN1–PS protein. Detection with an anti-His antibody (Sigma-Aldrich, Taufkirchen, Germany; SAB1305538) confirmed the presence of the GUN1–PS protein. The protein concentration was determined using the Qubit protein assay kit (Invitrogen, Thermo Fisher Scientific; Q33211), and the protein was used fresh or stored at −80°C for further use after addition of an equal volume of 50% glycerol.

For EMSA experiments, the indicated amounts of purified protein were used in the binding reactions. Each reaction consisted of 4 μl of 5× binding buffer (50 mM Tris–HCl [pH 7.5], 50 mM NaCl, 200 mM KCl, 5 mM MgCl_2_, 5 mM EDTA, 5 mM DTT, 0.25 mg/ml BSA, and 5% glycerol), the specified amounts of protein, and 2 μl of a 1 nM Cy5-labeled probe. For competitor assays, the indicated amount of competitor was added to the binding reaction. The reactions were incubated for 30 min at 23°C, followed by addition of 2 μl of 20% Ficoll 400 (v/v). The samples were then run on a 5% native polyacrylamide gel in a cold room at 4°C. The gel was preconditioned for 1 h at 60 V in 0.5× TBE containing 2.5% glycerol to remove any residual ammonium persulfate. One well was loaded with 1× Orange G loading buffer as an indicator. Gel electrophoresis was performed at 60 V until adequate separation was achieved. The Cy5 signal was then detected using a FUSION FX scanner (VILBER LOURMAT GmbH, Eberhardzell, Germany).

### RIP-seq and RT–qPCR

For RIP, we adapted a previously described method (Wang et al., 2022) with some modifications. Four-day-old seedlings grown on 1/2 MS medium were fixed with 1% formaldehyde for 15 min by vacuum infiltration. The fixation was stopped with 125 mM glycine for 5 min, again by vacuum infiltration. The seedlings were then washed four times with prechilled sterile ddH_2_0, ground to a fine powder with liquid nitrogen, and stored at −80°C for later use. Each ground plant sample (250 mg) was homogenized in 1 ml of RIP buffer. The composition of the RIP buffer was consistent with that of the original paper. Instead of preparing the beads–antibody conjugate, commercial GFP Trap Magnetic Agarose beads (gtma-20; ChromoTek) were used. Forty microliters of GFP-Trap was initially washed three times with 400 μl of RIP buffer and then incubated with 800 μl of cleared lysate for 2 h. The remaining steps for IP, RNA release, and extraction were performed following the previously outlined procedure (Wang et al., 2022). A western blot was performed for input, flow-through, and pull-down fractions of all samples with a GFP polyclonal antibody (Invitrogen; A6455). DNA contamination was removed using 2 U DNaseI (NEB; M0303S), and samples were then purified with the RNA Clean & Concentrator-5 Kit (Zymo).

For subsequent sequencing, the RNA was processed with the NEBNext Ultra II RNA Library Prep Kit from Illumina (NEB; E7770L). The libraries were then sequenced on an Illumina NextSeq 1000 system and analyzed on the Galaxy platform (Afgan et al., 2016). For RT–qPCR, 2 μl of purified RNA was reverse transcribed using the SuperScript IV Reverse Transcriptase Kit (Invitrogen, 18090050) with random hexamer priming. The cDNA synthesis reaction was performed under the following conditions: initial incubation at 23°C for 10 min, followed by reverse transcription at 55°C for 15 min for efficient cDNA synthesis. The reaction was then inactivated by heating at 80°C for 10 min. RT–PCR was performed on a Bio-Rad iQ5 real-time PCR instrument using SYBR Green Supermix (Bio-Rad; 1725274). All primer information is provided in Supplemental Table 7.

## Data and code availability

Sequencing data have been deposited in NCBI’s Gene Expression Omnibus (Edgar et al., 2002) and are accessible under GEO: GSE202931. Reads from experiments performed by Habermann et al. (2020), Zhao et al. (2019), and Xu et al. (2020) were retrieved from the NCBI Sequence Read Archive (SRA: PRJNA557616 and PRJNA432917, respectively) and Gene Expression Omnibus (GEO: GSE130337).

## Funding

Funding was provided by the 10.13039/501100001659Deutsche Forschungsgemeinschaft to C.S.-L., D.L., and T.K. (TRR175, projects A02, C01, and C05). Research in the Hua laboratory was supported by a US NSF CAREER award (MCB-1750361).

## Acknowledgments

We thank David Meinke for critical discussions, Michael Färberböck and Katrin Straßer for excellent technical assistance, Irma Racic for library preparation for RIP-seq, Helmut Blum and Stefan Krebs for sequencing the RIP libraries, and Eslam Abdel-Salam for help with statistical questions. No conflict of interest is declared.

## Author contributions

Conceptualization, Q.T., D.X., and T.K.; formal analysis and supervision, T.K.; investigation, Q.T., D.X., A.B., B.L., M.M.Y., T.M., Z.H., and T.K.; writing – original draft, T.K., with input from Z.H., C.S.-L., and D.L.; writing – review & editing, all authors; funding acquisition, C.S.-L., Z.H., D.L., and T.K.

## Supplemental information

Document S1. Supplemental Figures 1‒12 and Supplemental Table 7

Supplemental Table 1. Whole-genome transcript accumulation in 4-day-old *gun1W*, *gun1M*, and *gun1G* seedlings compared with the WT Data were analyzed with 3D RNA-Seq (see Methods). See Supplemental Table 2 for a separate, more detailed analysis of chloroplast transcripts incorporating the mapping of reads to inverted repeats.

Supplemental Table 2. Plastid transcript accumulation in 4-day-old *gun1W*, *gun1M*, and *gun1G* seedlings compared with the WT Data were analyzed with the DESeq2 program (see Methods).

Supplemental Table 3. Plastid transcript accumulation in seedlings treated with norflurazon (Habermann et al., 2020) or lincomycin (Xu et al., 2019a) Habermann et al. (2020) used four-day-old seedlings grown under continuous illumination (115 μmol photons m^−2^ s^−1^) at 22°C. Xu et al. (2020) used five-day-old seedlings grown under continuous illumination (100 μmol photons m^−2^ s^−1^) at 22°C. Data were analyzed with the DESeq2 program (see Methods).

Supplemental Table 4. Prediction of GUN1 target sites in the plastid genome As a search sequence, the least stringent sequence “AABBYSBYSAH” was used. “Matched sequence” indicates the prospective target sequence. Allowing U, C or G for the ambiguous B and only G for “G >> C” results in 25 potential target sites (column “Also in AABBYGBYGAH”). Allowing only U or C for the ambiguous Y and only G for “G >> C” results in 9 potential target sites (column “Also in AYYYGYYGAY”). Target sites in polycistrons are indicated by frames.

Supplemental Table 5. Analysis of RIP-seq data showing normalized read depths at each position in the chloroplast genome Read depths determined using SAMtools depth were multiplied by 1 million and then divided by the total number of reads mapped to the chloroplast genome in each sample.

Supplemental Table 6. RNA targets of GUN1 as determined by RIP-seq experiments Enriched transcripts were selected using the following selection criteria: >8-fold enrichment in both comparisons GUN1–GFP versus PP7L–GFP and GUN1–GFP versus Col-0, not too much variation between GUN1–GFP replicates (Tukey’s HSD test, *P* < 0.05). “Base mean” is defined as the average of the normalized count values, divided by size factors, taken over all samples, and is therefore an estimate of the expression level (low, medium, high) of a gene across all analyzed samples. The term “FeatureCounts” refers to the normalized count values. Column 2 shows the fold changes of transcript levels in *gun1-102* compared with Col-0. The *YCF1.1* transcript row is marked in gray because its sequence is part of the *YCF1.2* sequence.

Document S2. Article plus supplemental information
