# Supplementary material for: GENOMES UNCOUPLED PROTEIN1 binds to plastid RNAs and promotes their maturation
Source: Plant Commun. 2024 Aug 22;5(12):101069. doi: 10.1016/j.xplc.2024.101069 (PMC11671767; doi:10.1016/j.xplc.2024.101069)
Supplement: Document S1. Supplemental Figures 1‒12 and Supplemental Table 7 [file mmc1.pdf]

Plant Communications, Volume *5*

Supplemental information

GENOMES UNCOUPLED PROTEIN1 binds to plastid RNAs and promotes their maturation

Qian Tang, Duorong Xu, Benjamin Lenzen, Andreas Brachmann, Madhura M. Yapa, Paymon Doroodian, Christian Schmitz-Linneweber, Tatsuru Masuda, Zhihua Hua, Dario Leister, and Tatjana Kleine

Supplemental information

GENOMES UNCOUPLED PROTEIN1 binds to plastid RNAs and promotes their maturation

Qian Tang[a], Duorong Xu[a], Benjamin Lenzen[b], Andreas Brachmann[c], Madhura M Yapa[d], Paymon Doroodian[d], Christian Schmitz-Linneweber[b], Tatsuru Masuda[e], Zhihua Hua[d], Dario Leister[a], Tatjana Kleine[a,*]

[a]Plant Molecular Biology (Botany), Faculty of Biology, Ludwig-Maximilians-University München, 82152 Martinsried, Germany
[b]Molecular Genetics, Humboldt-University Berlin, Philippstr. 13, 10115 Berlin, Germany
[c]Biocenter of the LMU Munich, Genetics Section, Grosshaderner Str. 2-4, 82152 Planegg-Martinsried, Germany
[d]Department of Environmental and Plant Biology, Ohio University, Athens, Ohio 45701, United States
[e]Graduate School of Arts and Sciences, The University of Tokyo, Komaba, Meguro-ku, 153-8902 Tokyo, Japan

[*]Corresponding author:
Tatjana Kleine
e-mail: tatjana.kleine@lmu.de

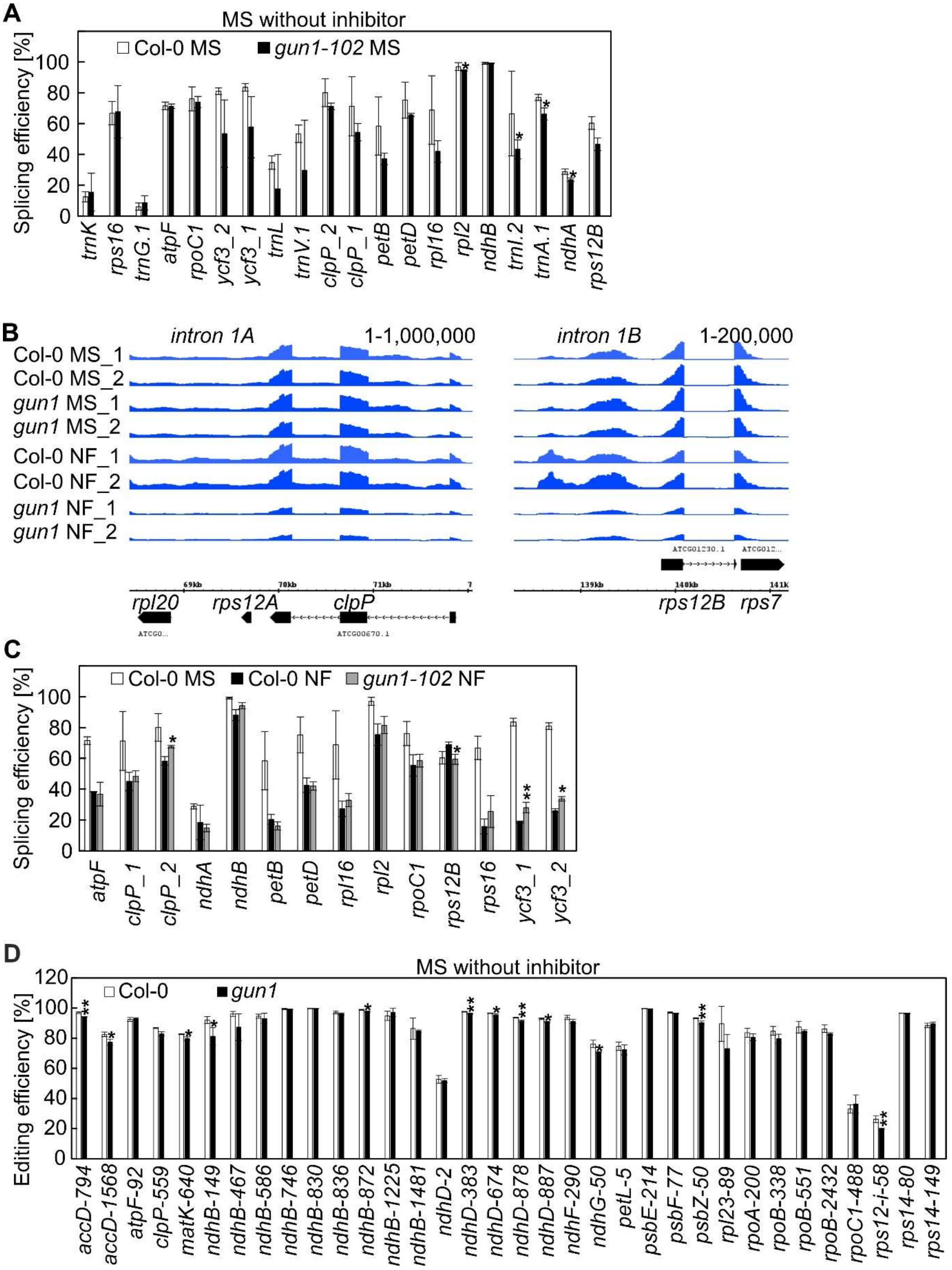

Supplemental Figure 1. GUN1 does not play a significant role in plastid RNA editing or splicing under normal growth conditions.

(A) RNA splicing efficiencies of 4-day-old Col-0 and *gun1-102* seedlings grown on MS were determined using previously published RNA-Seq data (Habermann et al., 2020). These sequencing data were generated to allow for detection of organellar transcripts. Mean values ±

SD were obtained from three independent experiments. Statistically significant differences between Col-0 MS and *gun1-102* MS are indicated (post-hoc Tukey HSD test; $*P < 0.05$).
(B) Snapshots across the *clpP* gene and intron 1B of *rps12B*. The read depths were visualized with the Integrated Genome Browser. Intron 1 of *rps12* is transcribed from two separate chromosomal regions: one downstream of *rps12A* and the other upstream of *rps12B*. They are then spliced together in trans. Therefore, we conducted a manual investigation of this intron using coverage files from the sequencing data.
(C) RNA splicing efficiencies of 4-day-old Col-0 and *gun1-102* seedlings grown on MS and norflurazon (NF) were determined using previously published RNA-Seq data (Habermann et al., 2020). These sequencing data were generated to allow for detection of organellar transcripts. Mean values ± SD were obtained from three independent experiments. Statistically significant differences between Col-0 NF and *gun1-102* NF are indicated (post-hoc Tukey HSD test; $*P < 0.05$, $**P < 0.01$). Due to the numerous modifications, structure and small size of tRNAs, the lncRNA-Seq library preparation method is not reliable for their detection. Hence, the splicing efficiency data for the six tRNA introns have to be viewed with caution and were excluded from further analysis.
(D) RNA editing efficiencies were calculated from data described in (A).

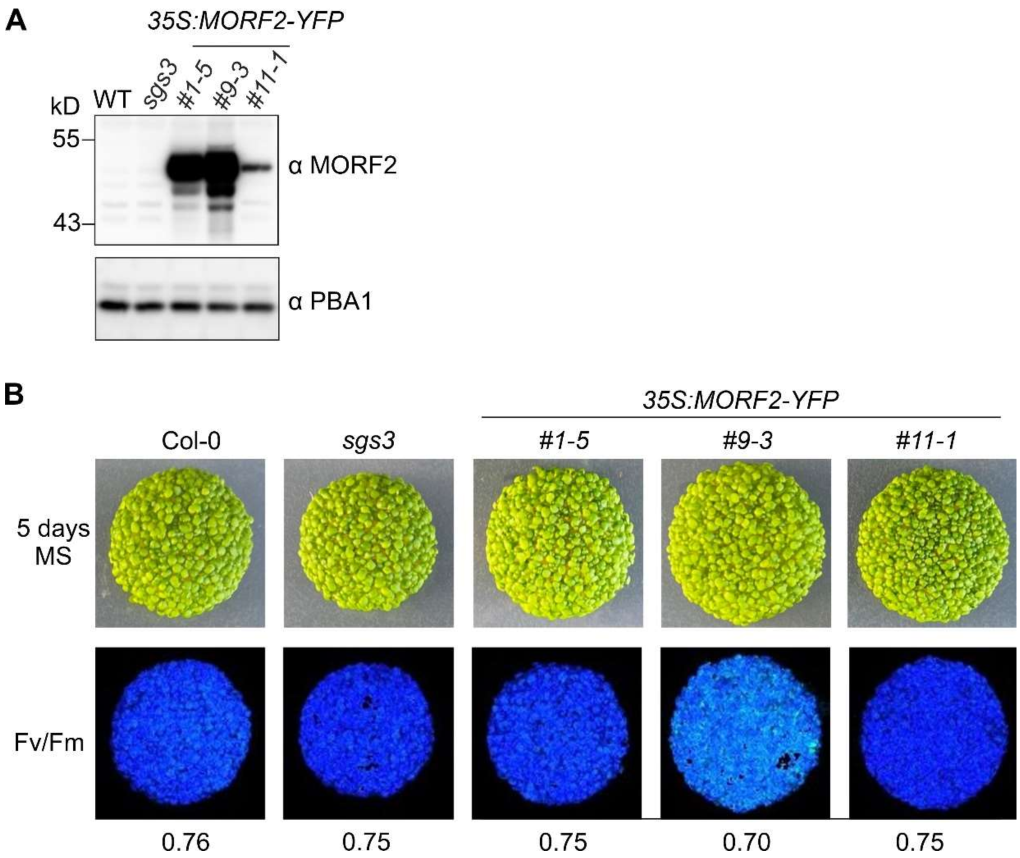

Supplemental Figure 2. Characterization of *35S:MORF2-YFP* lines.

(A) Immunoblot analysis showing protein levels of MORF2-YFP in three overexpression transgenic lines (*35S:MORF2-YFP*). Total proteins from 7-d-old seedlings were directly extracted in 2 x SDS sample buffer and denatured at 95°C for 6 min before being resolved on a 10% SDS-PAGE gel. MORF2-YFP was detected with an anti-MORF2 polyclonal antibody as described in (Yapa et al., 2023). The 20S proteasome subunit PBA1 was used to verify nearly equal loading of total protein. The three lines, *35S-MORF2-YFP #1-5*, *#9-3* and *#11-1*, were selected for further analysis as they exhibit *MORF2* overexpression to varying degrees.

(B) Phenotypes and Fv/Fm Imaging PAM pictures of 5-day-old Col-0, *sgs3-1* and *35S:MORF2-YFP* lines grown on MS medium without inhibitor supplementation.

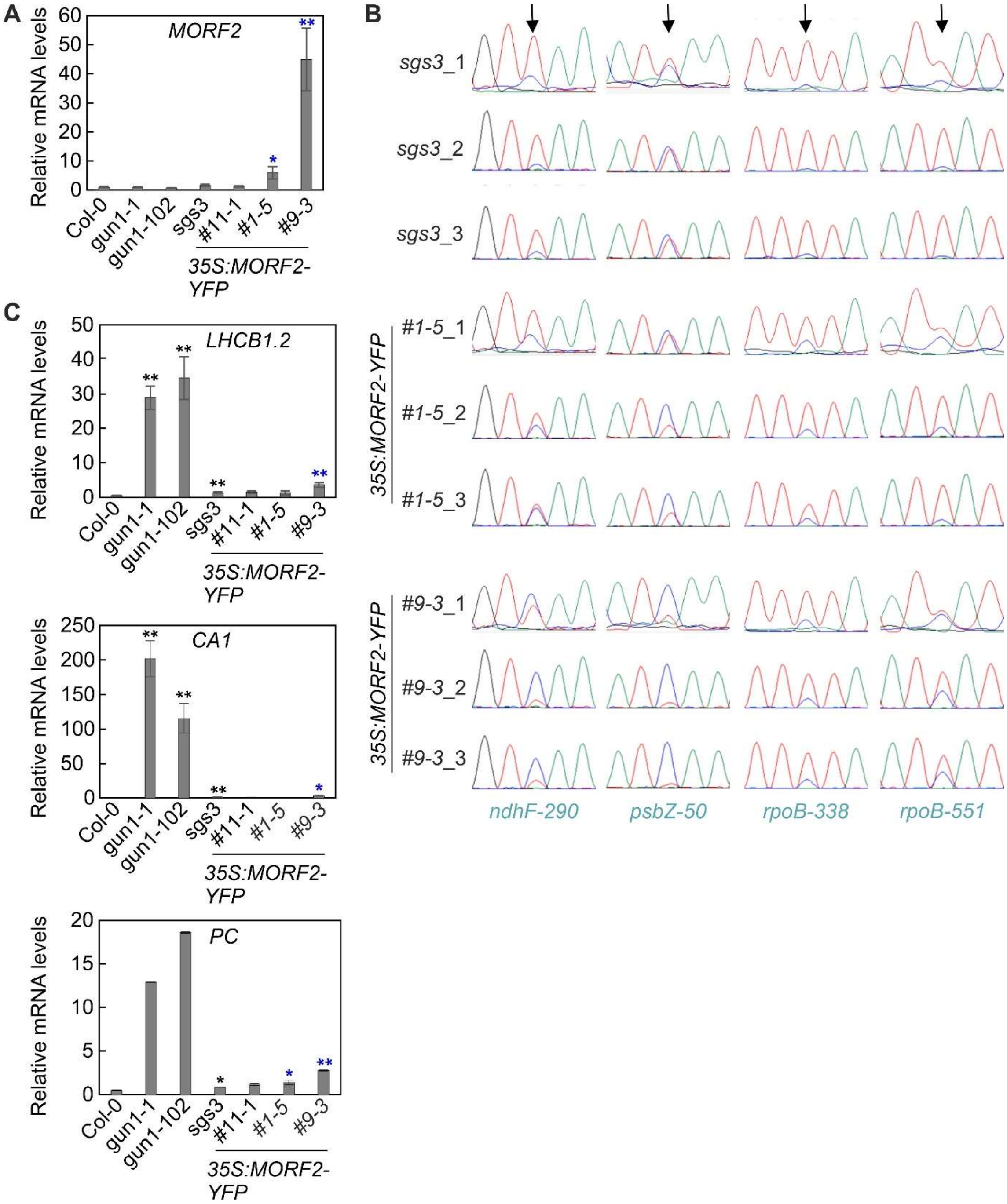

Supplemental Figure 3. Overexpression of MORF2 does not result in a significant *gun* phenotype.

(A) RT-qPCR of *MORF2* expression in 5-day-old seedlings grown under norflurazon (NF) conditions. The results were normalized to *AT4G36800*, which encodes a RUB1-conjugating enzyme (RCE1). Expression values are reported relative to the corresponding transcript levels in Col-0, which were set to 1. Mean values ± SE were derived from three independent experiments, each performed with three technical replicates per sample. Statistically significant differences (post-hoc Tukey HSD test; *$P < 0.05$; **$P < 0.01$) between Col-0, *gun1* and *sgs3*-

1 mutants are indicated by black asterisks, and those between *sgs3-1* and the *35S:MORF2-YFP* lines are indicated by blue asterisks.

(B) Seedlings were grown as in (A). Editing efficiency of selected sites was visualized by Sanger sequencing for three biological replicates.

(C) RT-qPCR of *LHCB1.2*, *CARBONIC ANHYDRASE 1* (*CA1*), and *PLASTOCYANIN* (*PC*) was performed using the identical cDNAs as in (A).

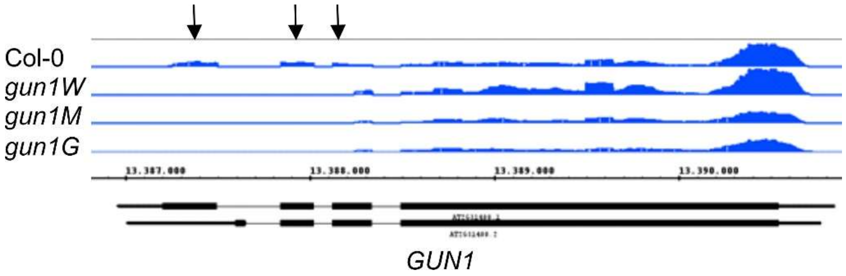

Supplemental Figure 4. Validation of RNA-Seq data and the *gun1-102* allele.
A snapshot across the *GUN1* gene is shown. The read depths were visualized with the Integrated Genome Browser. Arrows point to the absence of reads in a portion of exon 2 and the subsequent exons in *gun1W*, *gun1M*, and *gun1G*. The absence of transcription in a portion of exon 2 and subsequent exons of the *GUN1* gene was verified in all *gun1* mutant seedlings, confirming the T-DNA insertion in all *gun1* seedlings and validating the RNA-Seq data.

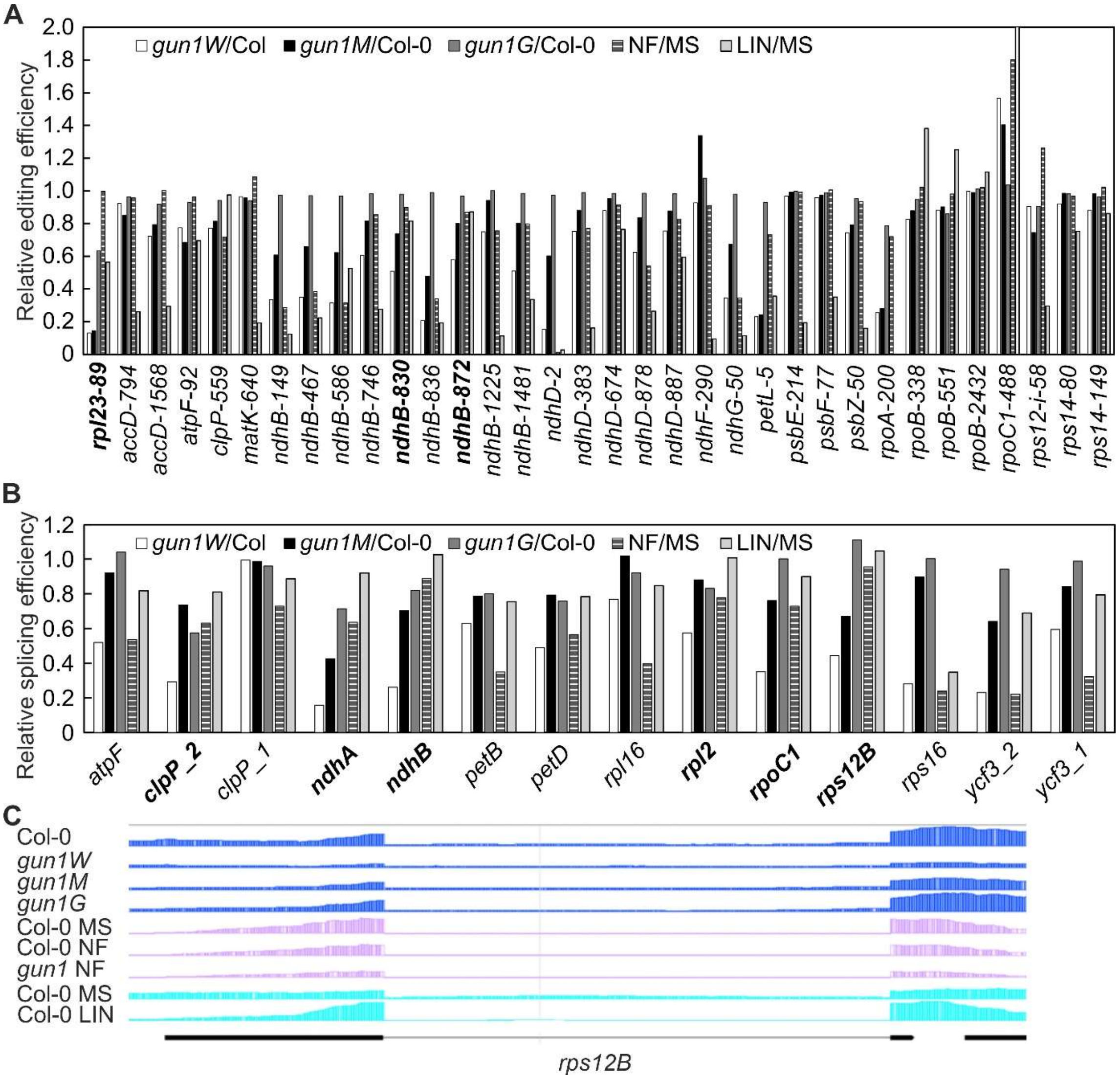

Supplemental Figure 5. Analysis of editing and splicing efficiencies of *gun1W*, *gun1M* and *gun1G* seedlings in comparison to seedlings grown on norflurazon (NF) and lincomycin (LIN).

A, B) RNA editing **(A)** and splicing **(B)** ratios of 4-day-old white (*gun1W*), marble (*gun1M*), and green (*gun1G*) *gun1-102* seedlings compared to Col-0 (Col), and ratios of seedlings grown on norflurazon (NF) or lincomycin (LIN) compared to seedlings grown on MS. The NF, LIN, and MS data were extracted from previously published RNA-Seq data (Habermann et al., 2020). We identified loci in which the relative ratio of editing or splicing was lower in *gun1W*/Col-0, progressively rescued in *gun1M*/Col-0 and *gun1G*/Col-0, and absent in NF/MS or LIN/MS. Concerning editing changes, the *ndhB-830* and *ndhB-872* editing sites were weak candidates, while *rpl23-89* was a stronger candidate. However, the editing efficiency of *rpl23* is reduced under stresses (Xu et al., 2023), speaking for a pleiotropic effect. Additionally, editing of *rpl23* was not progressively restored in *gun1M* seedlings. Regarding splicing alterations, potential affected loci included *ndhA*, *ndhB*, *rpoC1*, *rps12B*, *clpP_1* (no progressive rescue observed for *clpP_1*), and *rpl2* (weak; see fig. S7).

(C) Snapshots across the *rps12B* gene. The read depths were visualized with the Integrated Genome Browser. Splicing for *rps12B* was still observed, and notably, *rps12B* transcripts were largely decreased.

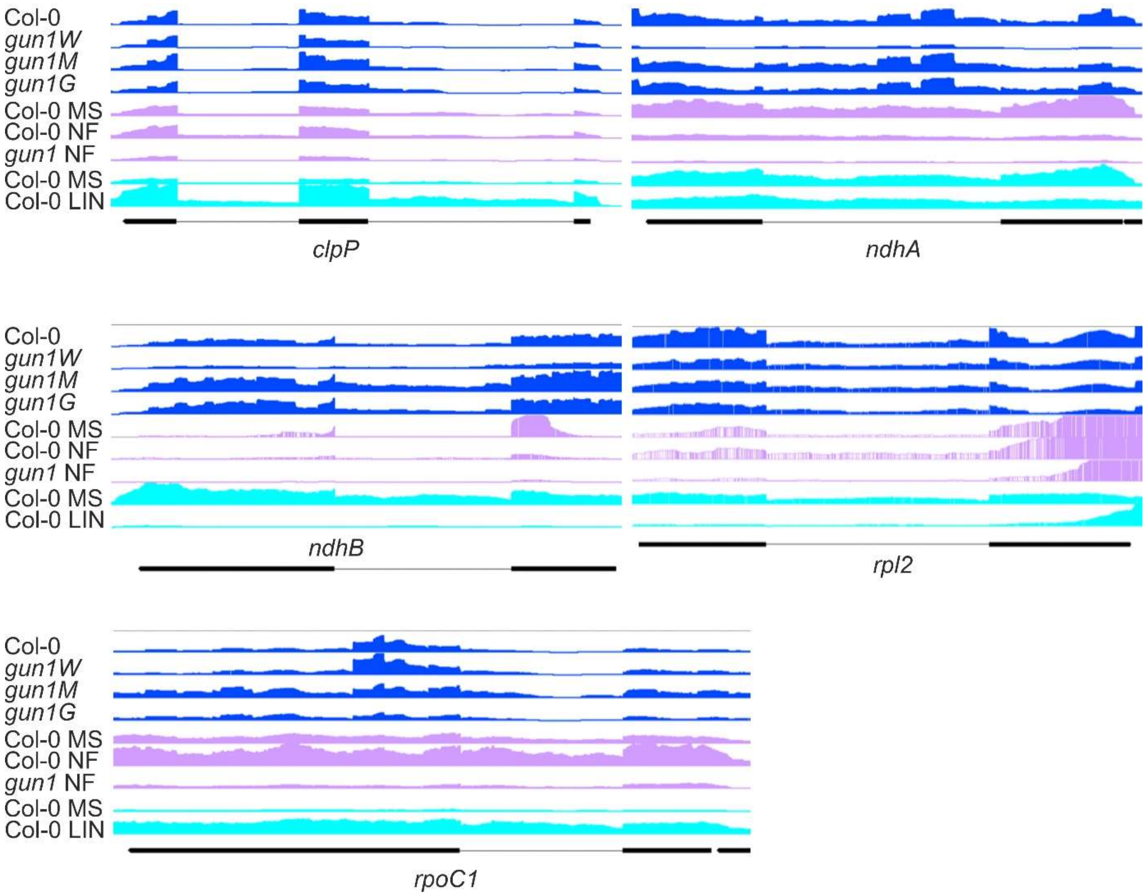

Supplemental Figure 6. Illustration of splicing behavior of selected transcripts. Snapshots across the *clpP*, *ndhA*, *ndhB*, rpl2, and *rpoC1* genes are shown. The read depths were visualized with the Integrated Genome Browser.

A

ID	Gene	*gun1W*	*gun1M*	*gun1G*	NF	LIN	Pred. target of GUN1
ATCG00360	*ycf3*	0.42	1.30	1.10	0.66	0.99	no
ATCG00380	*rps4*	0.48	1.05	0.96	0.83	0.79	no
ATCG00500	*accD*	0.46	0.85	0.80	0.65	0.71	no
ATCG00520	*ycf4*	0.31	0.94	1.04	0.50	0.79	yes
ATCG00640	*rpl33*	0.38	0.85	0.97	0.55	0.87	no
ATCG00660	*rpl20*	0.45	0.68	0.77	0.76	2.42	yes
ATCG00740	*rpoA*	0.28	0.74	1.03	0.99	1.85	no
ATCG00750	*rps11*	0.24	0.72	1.14	1.13	2.02	no
ATCG00760	*rpl36*	0.22	0.78	1.30	1.11	2.30	no
ATCG00770	*rps8*	0.17	0.50	0.99	1.21	2.37	no
ATCG00780	*rpl14*	0.15	0.43	0.99	0.74	1.79	no
ATCG00790	*rpl16*	0.13	0.32	0.77	0.73	1.70	no
ATCG00800	*rps3*	0.19	0.63	1.29	0.98	1.44	no
ATCG00810	*rpl22*	0.17	0.43	0.93	1.07	1.41	no
ATCG00820	*rps19*	0.25	0.42	0.58	1.12	1.53	no
ATCG01120	*rps15*	0.42	0.96	0.97	0.68	1.96	no

B

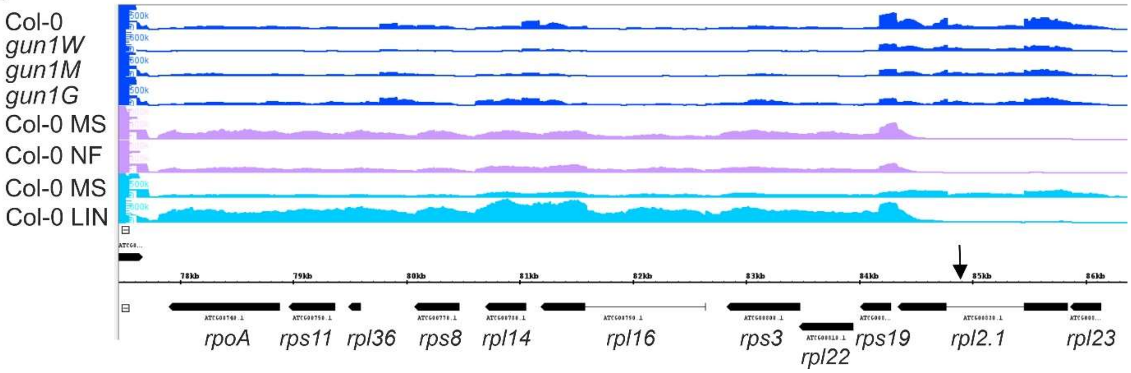

Supplemental Figure 7. GUN1 deficiency has a significant impact on the entire chloroplast transcriptome.

(A) Fold changes of transcripts that are exclusively reduced in *gun1W* are shown. n.d. denotes that these transcripts were not detected in the RNA-Seq analysis; red., reduced.

(B) Coverage plots depict the accumulation of reads across the indicated gene cluster.

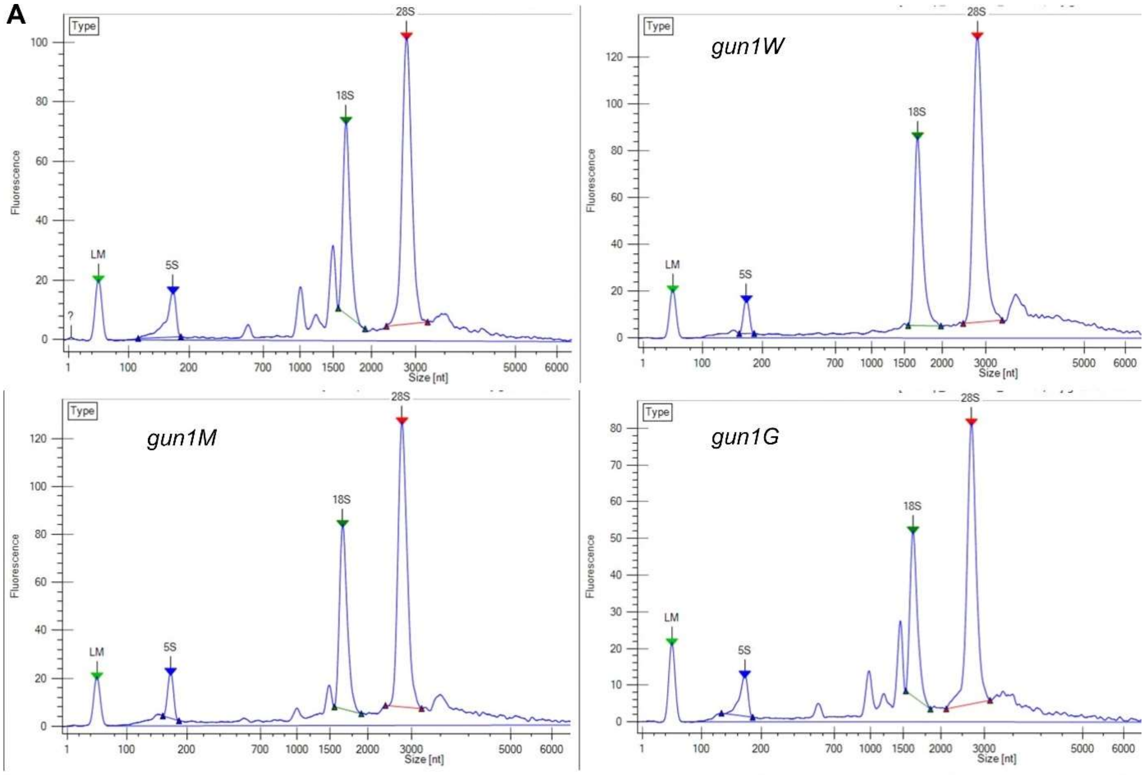

Supplemental Figure 8. Bioanalyzer profiles of total RNAs prepared with the RNA 6000 Nano Kit (Agilent).

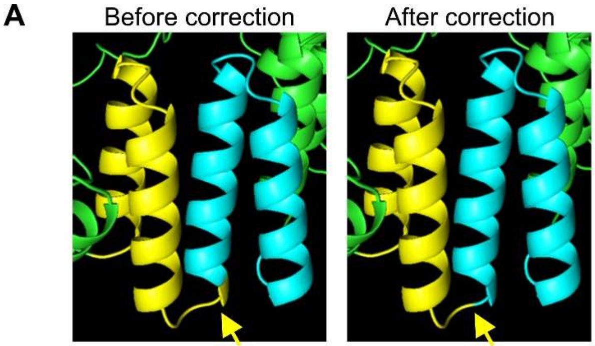

Supplemental Figure 9. PPR domain modeling of the GUN1 protein.

Section of PPR domain modeling of the GUN1 protein. The 12 predicted PPR domains of GUN1 by ScanProsite and the PPR CODE PREDICTION WEB SERVER (http://yinlab.hzau.edu.cn/pprcode; Yan et al., 2019) differ by a shift of one amino acid. Since the correct PPR code is crucial for determining the binding sequence, we investigated the structural configuration of the GUN1 protein by modeling with PyMOL. Each PPR domain was marked with a unique color for easy differentiation and visualization. Notably, within this color-coded scheme, helix b (yellow) of the initial PPR domain is observed to extend beyond (marked with an arrow) helix a (turquoise) of the subsequent PPR domain.

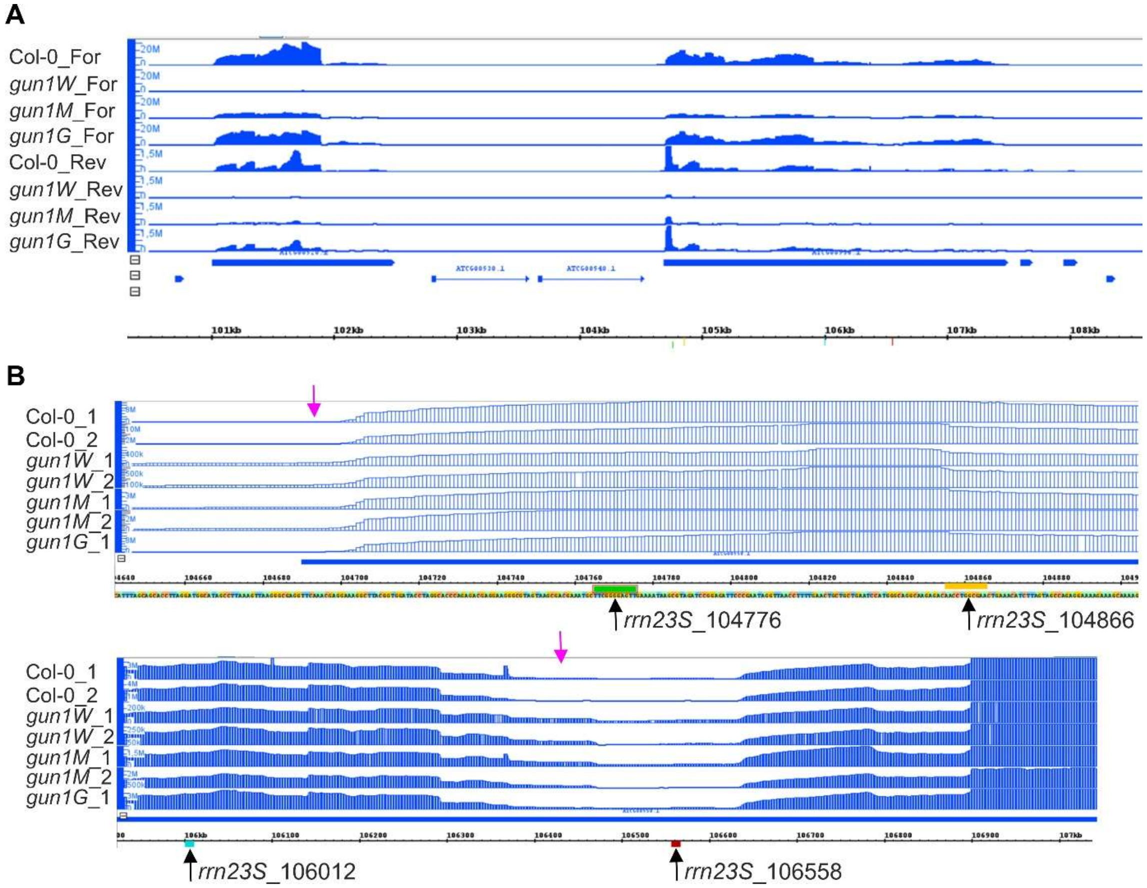

Supplemental Figure 10. Plastid ribosomal RNAs are significantly reduced in *gun1W* and *gun1M* seedlings.

(A) Read depths across the ribosomal operon were visualized using the Integrated Genome Browser. Note that this sequencing technique does not reliably capture tRNAs.

(B) Plots of RNA-Seq data produced without rRNA depletion depict a proportional increase in reads in *gun1W* and *gun1M* that map to 23S ribosomal RNA regions (magenta arrows) close to the four predicted GUN1 binding sites (marked with black arrows).

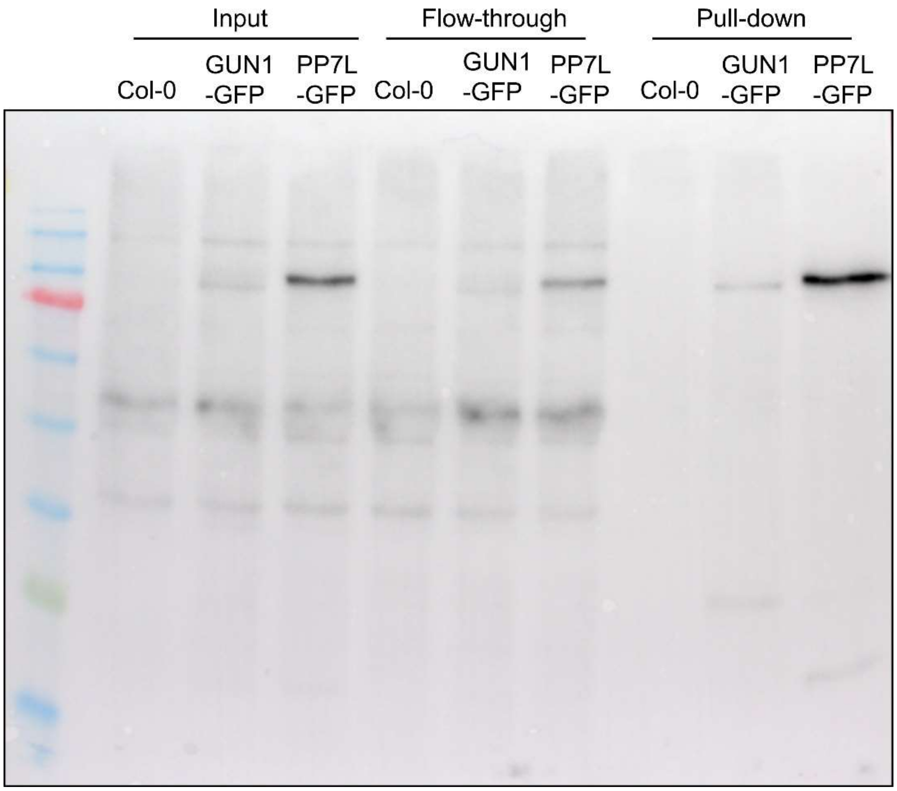

Supplemental Figure 11. Immunoblot analysis for the validation of the GUN1 RIP experiment.

Immunoblot analysis of proteins isolated from the input, flow-through and pellet (pull-down) fractions of the RIP experiment performed with proteins isolated from 4-day-old Col-0, *35S:GUN1-GFP*, and *35S:PP7L-GFP* seedlings. Proteins were fractionated by SDS-PAGE, and blots were probed with an antibody detecting GFP.

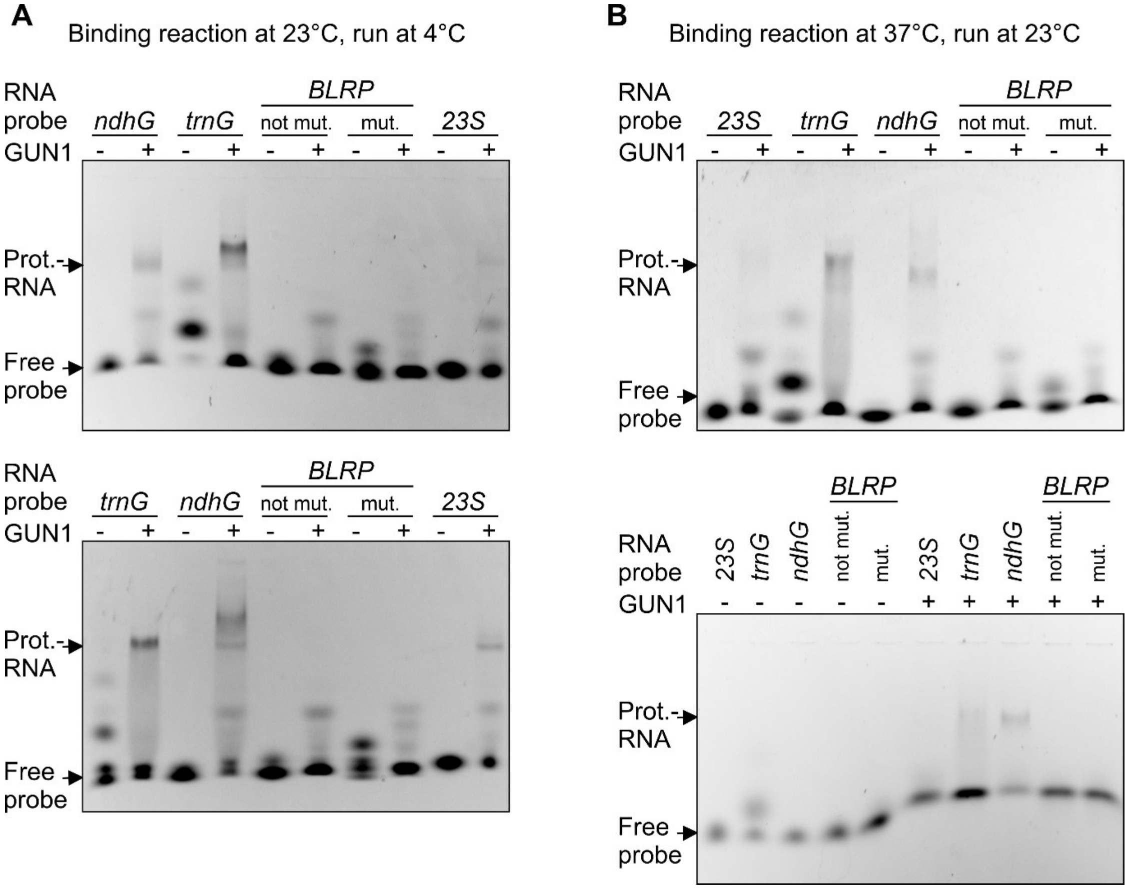

Supplemental Figure 12. EMSA experiments to test for GUN1 binding at the *BLRP*.
(A) EMSAs were performed with purified His-tagged GUN1 protein that was produced in *E. coli*. Aliquots (0, and 800 nM) of purified GUN1 protein were incubated with Cy5-labeled ssRNA probes representing the putative target sequences and a *BLRP* probe containing 10 mutated sites (mut.). Binding reactions were performed at 23°C, followed by electrophoresis on nondenaturing TBE polyacrylamide gels at 4°C.
(B) EMSAs were performed as in panel (A), but binding reactions were performed at 37°C, followed by electrophoresis on nondenaturing TBE polyacrylamide gels at 23°C.

Supplemental Table 7. Primers used in this study.

AGI number	Description	Primer sequence (from 5' to 3')
	**RT-qPCR**	
AT4G36800	"Housekeeping" gene RCE1	CTGTTCACGGAACCCAATTC
		GGAAAAAGGTCTGACCGACA
AT2G33430	MORF2	ATGGCTTTGCCTTTGTCTG
		AACCTGACCGGTTAGCTC
AT1G29910	LHCB1.2	CCGTGAGCTAGAAGTTATCC
		GTTTCCCAAGTAATCGAGTCC
AT3G01500	CA1	GAGAAATACGAAACCAACCCT
		ACATAAGCCCTTTGATCCCA
AT1G67100	PC1	CAACGCAGGGTTCCCACAT

	(primer combination used by Zhao et al. (2019))	CGCACAATAGAAACCGTAAGAGC
ATCG00730	petD	TCAAATACTTCGTACAGTGCCT
		TTGCTCCAATACCTAACCACAG
ATCG00790	rpl16	GACAAACCAGTTACAGTAAGACCT
		ACACCACCCATTTCATAAAGGA
ATCG00660	rpl20	CTTACACGAACTATGACTCAACAG
		TACGGCATTTATTCGAGTGATCC
ATCG00650	rps18	TTATCTAGACGGGTGAATAGAGTG
		TAAGACTAGTAGTTCTAGGAGTCG
ATCG00830	rpl2	CAGCATCATTGTGGTAAAGGT
		GCATATCGGTCAAAGGTAGG
AT2G31400	GUN1	GTCTTGAGTATATTGACTGGCTG
		GAGGCTGTAAAGCAAACGAC
ATCG00270	BLRP	ACCCATCGAATCATGACTATATCC
		AGAGATATCGACGGATTTCCT
**Editing detection by Sanger sequencing**		
ATCG00670	clpP_559	AATGATCCATCAACCCGC
		ATTGAACCGCTACAAGATC
ATCG00300	psbZ_50	GGGATTCGAACCCTCGATAG
		TCAAGTTCCATAAGTTCGACCC
ATCG00180	rpoC1_448 (same like Zhao et al. (2019))	TTTTCTTTTGCTAGGCCCATAA
		TTCGCAAATCTAAATCGGCT
ATCG00190	rpoB_338_and_551	TATCGGTTTATTGATCAGGG
		GCAGCTGCTAACACATCTC
ATCG00890	ndhB_467	TGCTTCTCTTCGATGGAAG
		TCCTTCGTATACGTCAGG
ATCG01010	ndhF_290	ACTGCCAGTTATCCAATAAAGAC
		TCATCCCTTTCATTCCACTTC
ATCG00065	rps12_-58	TGATTAGGTCATTTACCCTG
		AAATACAAGACAGCCAATCC
**Editing detection by amplicon sequencing**		
ATCG00670	clpP_559	TCTTGGAAGCGGAAGAATTACT
		TGAACCGCTACAAGATCAAC
ATCG00300	psbZ_50	CCACCAAGAAGACTAATCCAATCC
		GCTTTCCAATTGGCAGTTTTTG
ATCG00180	rpoC1_448	AGAAGGCCTAGTATACTGCGA
		TAATAATTCGCAAATCTAAATCG
ATCG00190	rpoB_551	GAAAACCAGTAGGAATATGC
		TCCCCACCTACACAAGAAAATTG
ATCG00890	ndhB_467	CCGATGGAGAGAAGAACCTATG
		TATCCAGATAATAGGTAGGAGC
ATCG01010	ndhF_290	AAAACCTTCGCCGCATGTGG

		ATCAGAACCAAAATCCCAACAG
**Northern blotting**		
AT1G29910	LHCB1.2	GACTTTCAGCTGATCCCGAG
		CGGTCCCTTACCAGTGACAA
ATCG01080	ndhG	GGATTTGCCTGGACCAATAC
		TGACGAGCCACAGAAATTGC
ATCG01130	ycf1.2	AGAGCCACATGGCGAGATTT
		TGGGACCACTCGGGAAATTG
**RIP-qPCR**		
ATCG00950	23S_104766 and 23S_104856	GATACCTAGGCACCCAGAGAC
		CTACTAAGATGTTTCAGTTCGCCA
ATCG00950	23S_106558	CGGAAGGTTAAGGAAGTTGG
		GGAATTTCGCTACCTTAGGAC
ATCG01080	ndhG	TCGATACGTCATGGTACGGG
		TGACGAGCCACAGAAATTGC
ATCG01130	ycf1.2	ATGTACCAATGGAGCCTGGA
		GGATCAAAGCCATTTCATCGT
ATCG00280	psbC (negative control)	ACTTCCCCACCTAGCCACTT
		AGCCCAAAACTGCAGAAGAA
ATCG00020	psbA (negative control)	TTTCCGGTGCCATTATTCCT
		TCATAAGGACCGCCGTTGTA
ATCG00360	ycf3 (negative control)	CGGATGTCGGCTCAATCTGAAGG
		AGGGGTTTCGTTCTAATGCCCGA
**EMSA**		
ATCG00950	23S_104856	aagagacaaccuggcgaacugaaac
ATCG01080	ndhG_118454	agaaaaaaaucuguugauaaaugaa
ATMG00190	trnG.1	auuugggaauuucuccauccaucau
Unspecific competitor	For 23S_104856 and ndhG_118454	auucuuauguggcagucucuaguccacuagcuuuug
	For trnG.1	gcaucugaauuucauaaccaaucucgau
ATCG00270	Kim_RNA1	ggaaauccgucgauaucucu
ATCG00270_mut	Kim_RNA3	ggaccgaaugaucuaucucu
